# RNA-Seq Identification of Peanut Callus-Specific Promoters and Evaluation of Base-Editing Efficiency

**DOI:** 10.3390/plants14152290

**Published:** 2025-07-25

**Authors:** Lulu Xue, Han Liu, Huanhuan Zhao, Pengyu Qu, Xiaona Li, Xiaobo Wang, Bingyan Huang, Ziqi Sun, Suoyi Han, Xiaodong Dai, Wenzhao Dong, Lei Shi, Xinyou Zhang

**Affiliations:** 1 College of Agronomy, Shenyang Agricultural University, Shenyang 110866, China; luluxue9331@163.com; 2 Institute of Crop Molecular Breeding, Henan Academy of Agricultural Sciences/Key Laboratory of Oil Crops in Huang-Huai-Hai Plains, Ministry of Agriculture/Henan Provincial Key Laboratory for Oil Crops Improvement/National and Provincial Joint Engineering Laboratory for Peanut Genetic Improvement, Zhengzhou 450002, China; liu1856215@163.com (H.L.); z2023huan@163.com (H.Z.); 17837860522@163.com (P.Q.); li12568xiaona@163.com (X.L.); biowangxb@163.com (X.W.); huangbingyan@aliyun.com (B.H.); sunziqi777@163.com (Z.S.); suoyi_han@126.com (S.H.); daixiaodong6666@163.com (X.D.); dongwzh@126.com (W.D.); 3 The Shennong Laboratory, Zhengzhou 450002, China

**Keywords:** peanut, callus-specific promoter, off-target reduction, cytosine base editing, genome editing efficiency

## Abstract

Prolonged expression of gene-editing components in CRISPR-modified plants can interfere with phenotypic analysis of target traits, increase the risk of off-target mutations, and lead to unnecessary metabolic burden. To mitigate these issues in peanut (*Arachis hypogaea* L.), callus-specific promoters were screened to restrict Cas9 expression to the callus stage, minimizing its activity in regenerated plants. In this study, six callus-specific genes in peanut were identified by mining RNA sequencing datasets and validating their expression profiles using quantitative reverse transcriptase PCR. The promoters of *Arahy.H0FE8D*, *Arahy.WT3AEF*, *Arahy.I20Q6X*, *Arahy.ELJ55T*, and *Arahy.N9CMH4* were cloned and assessed for their expression activity. Beta-glucuronidase (GUS) histochemical staining confirmed that all five promoters were functional in peanut callus. Further investigation revealed their ability to drive cytosine base editing via a deaminase-nCas9 fusion protein, with all promoters successfully inducing precise base substitutions in peanut. Notably, P_Ah-H0FE8D_, P_Ah-WT3AEF_, P_Ah-ELJ55T_, and P_Ah-N9CMH4_ exhibited comparable or higher editing efficiencies than the commonly used cauliflower mosaic virus 35S promoter. These findings provide valuable tools for improving the biosafety of CRISPR-based genome editing in peanut breeding programs.

## 1. Introduction

Peanut (*Arachis hypogaea* L.) is a globally important crop valued for both its nutritional and economic contributions as a source of food and oil. The advent of CRISPR/Cas-based genome editing technologies has revolutionized plant biological research and crop improvement by enabling precise and efficient modification of genes associated with key agronomic traits [1]. The adoption of CRISPR/Cas9 tools has significantly shortened the time required to develop new cultivars, from the traditional 8–10 years to just 4–6 years [2]. However, peanut plants typically produce only 20–40 seeds per plant, and regenerated T_0_ lines often yield even fewer viable seeds. This limitation makes it necessary to advance edited lines through at least two generations to effectively segregate transgenes. Notably, Xue et al. [3] observed the emergence of novel mutation types at target loci in T_1_ lines, suggesting ongoing activity of editing reagents retained from the original transformation vector. Prolonged expression of the sgRNA-Cas9 complex also heightens the risk of unintended off-target mutations [4,5,6].

Given that plant transformations typically begin with the callus stage, the use of callus-specific promoters to drive *Cas9* expression offers a promising strategy to restrict its activity to early development. This approach minimizes editing activity in regenerated plants, thereby reducing off-target effects and improving trait stability [7,8,9]. Genes exhibiting strong expression in callus tissue but only moderate or minimal activity in other tissues have been identified in maize [10], and their corresponding promoters were subsequently cloned and used to drive *Cas9* gene expression [8]. The pZmCTA1 callus-specific CRISPR/Cas9 system demonstrated significantly higher *Cas9* expression in callus, elevated mutation rates in transgenic callus, and reduced somatic mutations in regenerated plants, as compared to the CRISPR/Cas9 system driven by the maize *Ubiquitin* promoter [8]. Li et al. [11] employed a callus-specific promoter for *Cas9* expression and achieved not only high-efficiency gene editing but also high dual-gene homozygous mutation in cassava.

This study reports the identification of peanut callus-specific genes through RNA sequencing (RNA-seq) analysis followed by quantitative reverse transcriptase PCR (RT-qPCR) validation. Functionality evaluation of the corresponding promoters revealed that four out of the five tested could efficiently drive cytosine base editing at the P197 sites of *AhALS2-A* and *AhALS2-B* loci. These promoters yielded editing efficiencies comparable to or exceeding that of the widely used cauliflower mosaic virus 35S promoter-driven cytosine base editor (CBE). Collectively, these findings expanded the molecular toolkit for future peanut genome editing and provide versatile regulatory elements for enhancing precision and safety in future breeding programs.

## 2. Results

In this study, epicotyl segments from the peanut cultivar YH9326 were used as explants for callus induction. The initial explants were cultured on induction medium and subsequently cultured onto fresh medium every three weeks. After approximately five rounds of subculturing, the callus exhibited robust proliferation and developmental uniformity, making it suitable for downstream transformation experiments. As passaging continued, callus growth accelerated significantly, reaching peak vigor at the 10th to 11th subculture (Figure S1). However, by the 15th subculture and beyond, prolonged in vitro culture led to the accumulation of extensive somatic mutations, rendering the callus increasingly genomically unstable and therefore unsuitable for transformation [12,13,14].

**Figure 1:**
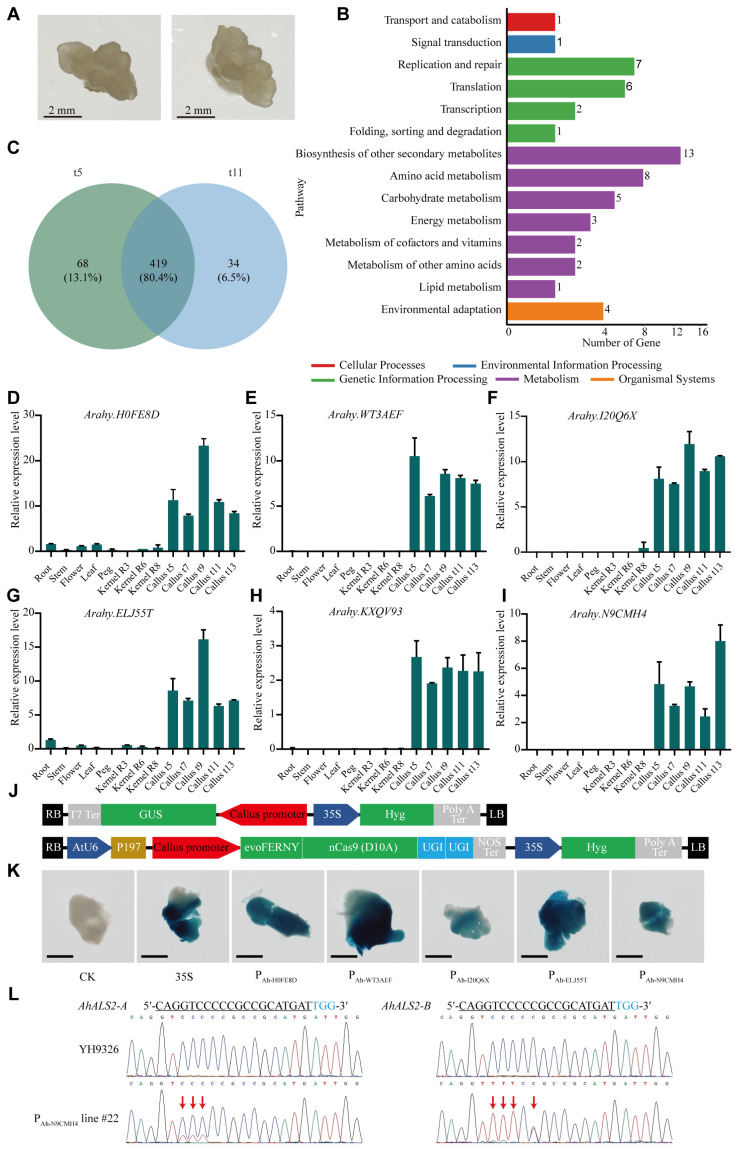
Identification of peanut callus-specific genes via RNA sequencing and validation. (**A**) Representative images of t5 and t11 peanut callus samples. (**B**) Kyoto encyclopedia of genes and genomes (KEGG) enrichment analysis of differentially expressed genes between t5 and t11 peanut callus samples. (**C**) Venn diagram showing the overlap of highly expressed genes found in both t5 and t11 peanut callus samples. (**D**–**I**) Tissue-specific expression profiles of candidate callus-specific genes: *Arahy.H0FE8D* (**D**), *Arahy.WT3AEF* (**E**), *Arahy.I20Q6X* (**F**), *Arahy.ELJ55T* (**G**), *Arahy.KXQV93* (**H**), and *Arahy.N9CMH4* (**I**) across various peanut tissues. R3, R6, and R8 represent reproductive stages: beginning pod, full seed, and harvest maturity, respectively. The housekeeping gene *AhADH3* was used as an internal control. Data are shown as mean ± SD from three biological replicates and four technical replicates. (**J**) Schematic diagram of the T-DNA constructs used for β-glucuronidase (*GUS)* reporter gene expression and cytosine base editing. T7 Ter: bacteriophage T7 RNA polymerase terminator; AtU6: Arabidopsis U6 promoter; 35S: 35S promoter of cauliflower mosaic virus; Callus promoter: P_Ah-H0FE8D_, P_Ah-WT3AEF_, P_Ah-I20Q6X_, P_Ah-ELJ55T_, or P_Ah-N9CMH4_; EvoFERNY: evolved cytidine deaminase; nCas9(D10A): Cas9 nickase; UGI: uracil-DNA glycosylase inhibitor; Hyg: hygromycin resistance gene; RB and LB: right and left T-DNA borders. (**K**) Histochemical staining of peanut callus transformed with *GUS* reporter gene driven by the 35S, P_Ah-H0FE8D_, P_Ah-WT3AEF_, P_Ah-I20Q6X_, P_Ah-ELJ55T_, and P_Ah-N9CMH4_ promoters, respectively. “CK” represents a control non-transformed YH9326 callus. Scale bar = 1 mm. (**L**) Representative Sanger sequencing chromatograms of the edited target sites of *AhALS2-A* and *AhALS2-B*. Target sequences are underlined, with the protospacer adjacent motif sequences and nucleotide mutations highlighted in blue and red, respectively.

In practice, callus at the 5th subculture typically represents the first cohort competent for genetic transformations, whereas callus at the 11th subculture serves as the most critical source for transformation due to its high productivity. To identify callus-specific promoters, RNA-seq was performed on callus samples from these two developmental stages, hereafter referred to as t5 and t11 callus, respectively (Figure 1A). The overall gene expression profiles between t5 and t11 samples were largely comparable, as reflected in the similar distribution of expression levels (Figure S2). However, a differential expression analysis revealed 231 genes that were significantly differentially expressed between t5 and t11 callus samples. Functional annotations of these differentially expressed genes (DEGs) indicated enrichment in categories such as DNA-binding transcription factor activity, acyltransferase activity, sequence-specific DNA binding, and transcription regulator activity (Figure S3). KEGG (Kyoto Encyclopedia of Genes and Genomes) enrichment analysis further showed that these DEGs were predominantly involved in key metabolism pathways, including amino acid metabolism, carbohydrate metabolism, biosynthesis of other secondary metabolites, as well as genetic information processing pathways such as DNA replication and repair, translation, and transcription (Figure 1B). These molecular signatures provide a basis for understanding the observed differences in growth dynamics between t5 and t11 callus.

To identify genes highly expressed in peanut callus, the top 400 genes with the highest FPKM (Fragments Per Kilobase per Million bases) values were selected from each of the six RNA-seq datasets [15]. This yielded 487 and 453 highly expressed genes from t5 and t11 peanut callus samples, respectively. Among these, 419 genes were consistently expressed in both t5 and t11 generations (Figure 1C, Table S1). To determine tissue specificity, the expression of these 419 genes across various peanut tissues was analyzed using the public peanut gene expression database (https://www.peanutbase.org/expression/expr_tissue_Hyp.html, accessed on 16 November 2024) [16]. Eighteen candidate genes exhibiting strong expression in callus while showing low or undetectable expression in other tissues were selected as candidate callus-specific genes (Table S2). RT-qPCR confirmed that *Arahy.H0FE8D*, *Arahy.WT3AEF*, *Arahy.I20Q6X*, and *Arahy.ELJ55T* were highly expressed in callus but showed minimal or no expression in root, stem, flower, leaf, peg, and developing kernels at R3 (beginning pod), R6 (full seed), and R8 (harvest maturity) stages [17] (Figure 1D–G). In contrast, *Arahy.KXQV93* and *Arahy.N9CMH4* exhibited lower expression in callus and were not detected in other tissues (Figure 1H,I). These six genes were thus identified as callus-specific candidates. Although *Arahy.KXQV93* and *Arahy.WT3AEF* shared high amino acid sequence similarity and similar expression patterns, *Arahy.KXQV93* exhibited lower expression levels in callus. As a result, *Arahy.KXQV93*, along with other genes showing notable expression in tissues beyond callus, was excluded from subsequent promoter functional studies (Figure S4).

Next, the promoters of *Arahy.H0FE8D*, *Arahy.WT3AEF*, *Arahy.I20Q6X*, *Arahy.ELJ55T*, and *Arahy.N9CMH4* were cloned and designated as P_Ah-H0FE8D_, P_Ah-WT3AEF_, P_Ah-I20Q6X_, P_Ah-ELJ55T_, and P_Ah-N9CMH4_, respectively (see Supplementary Materials and gene sequences). Cis-regulatory element analysis of the identified promoters was performed using the online tool PlantCARE (https://bioinformatics.psb.ugent.be/webtools/plantcare/html/, accessed on 6 January 2025) [18]. The analysis revealed that all five promoters contained core elements, including the TATA-box and CAAT-box, which are essential for transcription initiation [19,20]. In addition, each promoter harbored at least one light-responsive element, such as Box 4, AT1-motif or the GATA-motif, suggesting potential regulation by photomorphogenic cues. Furthermore, all promoters possessed inducible regulatory elements associated with hormone signaling pathways (ABRE, TCA-element, TGACG-motif, or P-box) and environmental stress responses (as-1, WUN-motif, ARE, TC-rich repeats, or STRE) (Figures S5–S9), indicating their responsiveness to diverse internal and external stimuli. To evaluate promoter activity, a series of expression vectors were constructed by fusing each promoter to the β-glucuronidase (*GUS*) reporter gene. The resulting constructs were subsequently introduced into peanuts to evaluate their transcriptional functionality in planta (Figure 1J). A histochemical assay of GUS activity confirmed that all five promoters were functional in peanut callus (Figure 1K). To further test their functional activity, CBEs constructs were constructed in which each promoter drove the expression of a deaminase-nCas9 fusion protein [21,22], using the P197 sites in *AhALS2-A* and *AhALS2-B* as the target sites (Figure 1J). A CBE driven by the commonly used 35S promoter (35S-CBE) served as the control.

The results showed that all tested promoters were capable of driving deaminase-nCas9 expression and inducing precise base substitutions or indel (insertion or deletion) mutations at the target site in peanut (Figure 1L and Figure S10, Table 1). The 35S-CBE yielded a 14.3% editing efficiency with a 7.1% homologous mutation rate. P_Ah-I20Q6X_-CBE achieved a lower editing efficiency of 7.1%. In contrast, P_Ah-WT3AEF_-CBE matched the 35S promoter in overall editing efficiency (14.3%) but exhibited a higher homozygous mutation rate of 14.3%. Notably, CBEs driven by P_Ah-H0FE8D_, P_Ah-ELJ55T_, and P_Ah-N9CMH4_ outperformed the 35S-CBE in editing efficiency while also achieving equal or greater homozygous mutation rates.

Off-target analysis revealed that all six promoter-driven base editors exhibited moderate or high editing efficiency at the *AhALS1* P197 site, which differs from the *AhALS2* target site by a single nucleotide mismatch (Tables S3 and S4, Figure S11). Notably, P_Ah-ELJ55T_-evoCBE demonstrated 20% editing efficiency at an off-target site with four mismatches relative to the original target sequence, indicating that this promoter can drive strong deaminase-nCas9 activity even under conditions of reduced sequence specificity.

## 3. Discussion

The editing efficiency of a CRISPR/Cas construct is largely dependent on the quantity of editor protein produced in individual transformed cells, with elevated expression leading to higher editing rates [7]. However, extended expression of *Cas9* nuclease gene can induce undesired off-target mutations [23,24]. Editing systems with conditionally activated Cas9 proteins can greatly reduce these effects [25,26,27]. Through the use of transient delivery methods or environmentally controlled activation, a variety of inducible genome editing systems have been developed to minimize off-target effects [28]. In plant bioengineering, spatiotemporal expression of *Cas9* can be achieved by using specific promoters. Ji et al. [29] employed two virus-inducible promoters for Cas9 expression and the two corresponding genome-editing constructs were transformed into *Arabidopsis*. No off-target mutations were observed in the transgenic plants either before or after virus infection, distinctly contrasting with plants in which continuous *Cas9* expression was maintained. Similarly, a heat-shock-inducible promoter-driven CRISPR/Cas9 system has been shown to produce transgenic rice lines with significantly lower off-target mutation rates compared to those generated using a constitutively expressed editing system [30].

Given that plant transformations typically commence at the callus stage, employing callus-specific promoters to temporally and spatially confine Cas9 activity is anticipated to substantially reduce off-target mutagenesis in regenerated mutant plants. This premise was corroborated by prior studies demonstrating that T_1_ plants generated using callus-specific CRISPR/Cas9 constructs exhibited significantly fewer novel mutations at target loci compared to plants engineered with constitutive maize *Ubiquitin* promoter-driven systems in maize [8]. Similar callus-specific promoters have also been successfully cloned and used in genetic transformation systems for rice [31] and cassava [11], demonstrating their broader applicability across diverse crop species.

To minimize unintended edits and off-target effects in regenerated peanut lines, peanut callus-specific genes were identified via RNA-seq analysis and validated through RT-qPCR (Figure 1D–I). As shown in Table S2, *Arahy.H0FE8D*, *Arahy.I20Q6X*, and *Arahy.N9CMH4* encode proteins with uncharacterized functions. *Arahy.KXQV93* and *Arahy.WT3AEF* encode Early Nodulin-93 proteins, which have been implicated in nodule development [32], nitrogen fixation [33], and nitrogen use efficiency [34]. *Arahy.ELJ55T* encodes an Enolase, a key enzyme in the glycolysis metabolic pathway [35]. Cis-element analysis of P_Ah-H0FE8D_, P_Ah-WT3AEF_, P_Ah-I20Q6X_, P_Ah-ELJ55T_, and P_Ah-N9CMH4_ revealed that all promoters contained essential transcription initiation elements, including TATA-box and CAAT-box. Additionally, various inducible elements responsive to light, hormones, biotic, or abiotic stresses were identified, suggesting that the activity of these genes may be regulated by diverse environmental and endogenous signals. Notably, Box-4 (a light-responsive element) and ARE (anaerobic responsive elements, a cis-acting regulatory element essential for the anaerobic induction) were present in all five promoters. Further investigations into the functional roles of these genes, as well as detailed promoter motif analyses, are warranted to elucidate the molecular mechanisms underlying their callus-specific expression.

Functional characterization revealed that four of these promoters exhibited base-editing efficiencies comparable to, or surpassing, the widely utilized 35S promoter. Interestingly, when used to drive CBEs, promoters exhibiting strong transcriptional activity in planta did not consistently translate into superior editing outcomes. For instance, although P_Ah-N9CMH4_ exhibited relatively modest expression levels, it achieved the highest editing efficiency among all tested promoters. Likewise, P_Ah-WT3AEF_ and P_Ah-I20Q6X_ showed comparable gene expression levels, yet the former mediated nearly double the editing efficiency of the latter. These observations suggest that editing efficiency is not solely dictated by promoter strength but may also be influenced by factors such as transcript stability, chromatin accessibility, and spatial–temporal expression dynamics. Given that particle bombardment frequently results in high transgene copy numbers [36], and that this study evaluated editing at a limited number of loci, further validation is warranted. Comprehensive assessment of these promoters across additional genomic loci and within diverse editing platforms, including prime editing, and Cas9- or Cas12-based gene knockout systems, will be essential to fully establish their utility and reliability in precision breeding applications.

Notably, all CBEs tested in this study generated a high proportion of indel mutations at the target site (Table 1, Figure S10). The occurrence of CBE-induced indels is likely attributable to DNA double-strand breaks, which may result from the synergistic action of high deaminase activity, endogenous uracil-DNA glycosylase, and nCas9-mediated DNA strand nicking [37]. The frequency of indel formation is known to vary across different genomic loci [21]. The artificially evolved deaminase evoFERNY, used in this study, has been previously shown to induce higher indel rates compared to the wild-type APOBEC1 deaminase in both mammalian cells [21] and rice [22]. Therefore, it is plausible that the elevated indel frequencies observed here are primarily a consequence of using the evoFERNY variant. Further investigation is warranted to test this hypothesis and to evaluate whether alternative deaminase could reduce unintended mutagenesis while maintaining high-editing precision.

Previous studies have shown that the truncation of promoter sequences can significantly alter their expression patterns and transcriptional strength [38]. In this study, the approximately 2000 bp upstream of the start codon were assumed to represent the native promoter regions of the selected genes. However, these cloned putative promoters may not fully ensure callus-specific expression of downstream genes [8]. Therefore, comprehensive characterization of promoter activity across multiple plant tissues in regenerated edited lines is necessary. Such analysis should be followed by rational extension or truncation of the promoter sequence to optimize both specificity and efficiency prior to broader application in plant transformation systems.

Significant off-target effects were observed in T_0_ transgenic callus lines generated using all tested CBEs, likely resulting from the low specificity of the selected sgRNA. As shown in Table S4, the most off-target edits occurred at the loci with a single-nucleotide mismatch relative to the sgRNA sequence. These findings highlight the critical importance of designing highly specific sgRNAs to minimize off-target activity and enhance genome editing precision [39,40].

Overall, this targeted promoter strategy offers significant potential for enhancing trait stability and minimizing off-target effects and can be adapted for genome editing applications in other economically important crop species.

## 4. Materials and Methods

### 4.1. Plant Materials

The peanut (*A. hypogaea*) variety YH9326 (also known as Yuhua 9326), developed by the Henan Academy of Agricultural Sciences (Zhengzhou, Henan, China), was used in this study. This cultivar is recognized for its high oil content and early maturity, making it a widely adopted peanut variety in the region.

### 4.2. RNA-Seq and Data Analysis

Total RNA was isolated from t5 and t11 peanut callus using a plant RNA extraction kit (Takara, Tokyo, Japan), following the manufacturer’s instructions. Library preparation and RNA-seq were performed by Wuhan Frasergen Bioinformatics Co., Ltd. (Wuhan, Hubei, China). DEGs were identified using DESeq2 v1.22.2 [41] with a fold change > 2 and a false discovery rate (FDR) < 0.05. Identified DEGs were further subjected to Gene Ontology (GO) and KEGG enrichment analysis.

### 4.3. Screening of Callus-Specific Genes in Peanut

Raw read counts from RNA-seq samples encompassing 22 different tissues and developmental stages were downloaded from the PeanutBase website [16]. The expression profiles of selected candidate genes were examined across these tissues. Genes showing high expression in callus but undetectable or significantly lower expression in these tissues were identified as peanut callus-specific genes.

### 4.4. Reverse Transcription and RT-qPCR

Total RNA was isolated from peanut callus (t5, t7, t9, t11, and t13), root, stem, flower, leaf, and peg (R2 stage, beginning peg), as well as kernels at reproductive stages R3 (beginning pod), R6 (full seed), and R8 (harvest maturity) of YH9326 plants [17]. RNA extraction was performed using a plant RNA extraction kit (Takara), following the manufacturer’s instructions. The developmental stages were defined according to [17]. First-strand cDNA was synthesized from 1 μg of total RNA using the PrimeScript^TM^ RT reagent kit (Takara) in a 20-μL reaction volume. RT-qPCR was performed using PowerUp^TM^ SYBR^TM^ Green Master Mix (ThermoFisher, Waltham, MA, USA) on an Applied Biosystems™ QuantStudio™ 5 Real-Time PCR system (ThermoFisher). *AhADH3* was used as the internal reference gene [42]. Each sample was analyzed with three biological replicates and four technical replicates. Relative gene expression levels were calculated using the 2^−ΔCt^ method [43]. Primer sequences used in RT-qPCR are listed in Table S5.

### 4.5. Cloning of Promoters and Cis-Element Analysis

Promoter sequences of peanut callus-specific genes were amplified using PrimeSTAR GXL DNA Polymerase (Takara) and specific primers (Table S5), with genomic DNA of wild-type YH9326 serving as the template. Each PCR reaction was prepared in a total volume of 50 μL, containing 10 μL buffer (Takara), 4 μL of dNTP mixture (2.5 mM, Takara), 0.3 μL each of forward and reverse primers, 2 μL of template DNA, 1 μL of DNA polymerase (Takara), and 32.4 μL of ddH_2_O. The amplification protocol included an initial denaturation at 94 °C for 30 s, followed by 30 cycles of 98 °C for 10 s, 55 °C for 15 s, and 68 °C for 2 min, with a final extension at 72 °C for 5 min. The PCR product was subjected to Sanger sequencing (Sangon Biotech, Shanghai, China). Cis-elements within each promoter were assessed using the online tool PlantCARE (https://bioinformatics.psb.ugent.be/webtools/plantcare/html/) [18].

### 4.6. Vector Construction and Peanut Transformation

The *GUS*-overexpression vector (preserved by our lab) was digested with *Sbf* I (ThermoFisher) and *Nco* I (ThermoFisher) to remove the P_Ah42CD1_ promoter driving the *GUS* reporter gene. The linearized product was resolved on a 1% agarose gel and purified using the FastPure Gel DNA Extraction Mini Kit (Vazyme, Nanjing, Jiangsu, China). Peanut callus-specific promoter sequences were amplified using primers containing 15 bp overlaps complementary to the vector ends. The resulting PCR products were inserted into the linearized vector using the In-Fusion^®^ cloning kit (Takara), resulting in five distinct *GUS*-overexpressing constructs driven by callus-specific promoters. An expression vector with the *GUS* gene driven by the 35S promoter served as the control.

The CBE vector (Weimi Biotechnology, Nanjing, China) was digested with *Bsa* I (ThermoFisher) for 1 h to introduce spacer sequences. The linearized product was purified using the same gel extraction method. Primers SG-P197-F and SG-P197-R were diluted to 10 OD in an annealing buffer (Solarbio, Beijing, China). A 5 μL aliquot of each primer was mixed in a PCR tube and subjected to an annealing program of 95 °C for 5 min, followed by a gradual cooling to 25 °C at a rate of 0.1 °C/s. The annealed product was directly ligated into the linearized CBE vector using T4 DNA ligase (Takara), generating the 35S-CBE construct. To replace the 35S promoter with peanut callus-specific promoters, the 35S-CBE construct was digested with *Sbf* I (ThermoFisher) and *Kpn* I (ThermoFisher) to excise the 35S promoter upstream of the deaminase-nCas9 fusion gene. The digestion product was purified using the same gel extraction method. Peanut callus-specific promoter sequences were amplified using primers containing 15 bp overlaps complementary to the vector ends. The resulting PCR products were inserted into the linearized vector using the In-Fusion^®^ cloning kit (Takara), resulting in five CBE constructs driven by callus-specific promoters. All recombinant CBE constructs, including 35S-CBE, were introduced into peanut callus via particle bombardment. Following transformation, peanut callus was placed on Murashige and Skoog (MS) medium [44], supplemented with 33 mg/L glutamine and 20 mg/L hygromycin, and incubated at 28 °C in darkness. The hygromycin-resistant calluses that emerged were further transferred to fresh induction medium without hygromycin for subculturing at 3-week intervals. After two to three rounds of subculture, the hygromycin-resistant calluses were collected for genotyping or GUS histochemical staining. Primer sequences used for promoter amplification are listed in Table S5.

### 4.7. Promoter Expression Activity Analysis

GUS histochemical staining was used to evaluate the transcriptional activity of peanut callus-specific promoters. Briefly, hygromycin-resistant calluses were submerged in GUS staining solution (Coolaber, Beijing, China) and incubated overnight at 37 °C to allow enzymatic color development. Following incubation, the samples were thoroughly washed with ethanol and GUS stain was examined under a dissecting microscope (SOPTOP, Yuyao, China).

### 4.8. Mutation Type Identification and Off-Target Detection

Genomic DNA was extracted from 24 to 28 randomly selected hygromycin-resistant lines by using the CTAB method [45]. Target sequences were amplified by PCR and the resulting products were sent for genotyping using the Hi-TOM (High-throughput Tracking Of Mutations) platform (China National Rice Research Institute, Hangzhou, Zhejiang, China) [46]. Sequencing reads with a frequency below 1% were excluded from analysis. The mutation type at each target locus was determined based on the mutation frequency, calculated as the number of edited reads divided by the total number of reads for the locus. A locus was classified as homozygously mutated if the mutation rate exceeded 90%, heterozygously mutated if the rate ranged between 20% and 90%, and chimeric if the mutation rate was below 20%. A line was considered an edited line if it contained at least one homologous or heterozygous mutation at either the *AhALS2-A* or *AhALS2-B* locus. Similarly, a line was categorized as homozygously mutated if at least one homologous mutation was detected at either of the two loci. The same classification approach was applied to identify lines carrying indels. It should be noted that when two or more mutation types were detected at a single locus, the mutation frequency was calculated based on the cumulative number of edited reads corresponding to all observed mutation types.

To assess potential off-target effects, candidate off-target sites were predicted using the CRISPR-P 2.0 online tool (http://crispr.hzau.edu.cn/CRISPR2/, accessed on 9 May 2025) [39]. The three sites with the highest off-target scores were selected for validation. Genomic DNA from edited lines was used as a template for PCR amplification of these regions, and the resulting amplicons were analyzed by Sanger sequencing (Sangon Biotech) to detect any unintended mutations.

## 5. Conclusions

Taken together, six peanut callus-specific genes were identified by mining RNA-seq data, followed by RT-qPCR validation. Promoter activity assays revealed that P_Ah-H0FE8D_, P_Ah-WT3AEF_, P_Ah-I20Q6X_, P_Ah-ELJ55T_, and P_Ah-N9CMH4_ were all functionally active in peanut callus. Notably, P_Ah-H0FE8D_, P_Ah-WT3AEF_, P_Ah-ELJ55T_, and P_Ah-N9CMH4_ drove base editing with efficiencies comparable to or exceeding that of the 35S promoter. These findings broaden the genome editing toolkit for peanuts and facilitate the application of callus-specific promoters in genetic improvement efforts.

## Figures and Tables

**Table 1:** Editing efficiencies of cytosine base editors (CBEs) driven by peanut callus-specific promoters.

Promoter-CBE Construct	Number of Genotyped Lines	Number of Edited Lines	Number of Homozygous Lines	Editing Efficiency (%)	Homozygous Rate (%)	Indel Rate (%)
35S-CBE	28	4	2	14.3	7.1	7.1
P_Ah-H0FE8D_-CBE	28	6	2	21.4	7.1	10.7
P_Ah-WT3AEF_-CBE	28	4	4	14.3	14.3	7.1
P_Ah-I20Q6X_-CBE	28	2	0	7.1	0	7.1
P_Ah-ELJ55T_-CBE	24	5	3	20.8	12.5	12.5
P_Ah-N9CMH4_-CBE	28	8	3	28.6	10.7	10.7

Note: Editing efficiency = (edited lines/genotyped lines) × 100%. Homozygous rate = (homozygous lines/genotyped lines) × 100%; Indel, insertion or deletion. Indel rate = (lines with indel mutations/genotyped lines) × 100%.

## Data Availability

The original RNA-seq data have been uploaded to the NCBI_SRA database (PRJNA1264070) (https://www.ncbi.nlm.nih.gov/sra/PRJNA1264070, accessed on 18 May 2025).

## Supplementary Materials

The following supporting information can be downloaded at: https://www.mdpi.com/article/10.3390/plants14152290/s1, Figure S1: Representative pictures of peanut callus on induction medium after 2 (a), 5 (b), and 11 (c) subcultures; Figure S2: Stacked bar chart showing the distribution of gene expression levels in t5 and t11 peanut callus samples; Figure S3: Gene Ontology (GO) enrichment analysis of the differentially expressed genes (DEGs) between t5 and t11 peanut callus samples; Figure S4: Relative expression levels of peanut callus-specific candidate genes across various tissues; Figure S5: Cis-regulatory element analysis of DNA sequence of P_Ah-H0FE8D_; Figure S6: Cis-regulatory element analysis of DNA sequence of P_Ah-WT3AEF_; Figure S7: Cis-regulatory element analysis of DNA sequence of P_Ah-I20Q6X_; Figure S8: Cis-regulatory element analysis of DNA sequence of P_Ah-ELJ55T_; Figure S9: Cis-regulatory element analysis of DNA sequence of P_Ah-N9CMH4_; Figure S10: Representative Sanger sequencing chromatograms of the edited target sites of *AhALS2-A* and *AhALS2-B*; Figure S11: Representative Sanger sequencing chromatograms of the off-target sites in edited lines; Table S1: Highly expressed genes in peanut callus; Table S2: Peanut callus-specific candidate genes; Table S3: Off-target site prediction of P197 target site; Table S4: Off-target analysis of peanut callus-specific promoters; Table S5: Primers used in this study; Gene sequences: nucleotide sequences of the five tested peanut callus-specific promoters.
