# Supplementary material for: RNA-Seq Identification of Peanut Callus-Specific Promoters and Evaluation of Base-Editing Efficiency
Source: Plants (Basel). 2025 Jul 25;14(15):2290. doi: 10.3390/plants14152290 (PMC12348465; doi:10.3390/plants14152290)
Supplement: Supplementary file 1 [file plants-14-02290-s001.zip › Supplementary Materials.pdf]

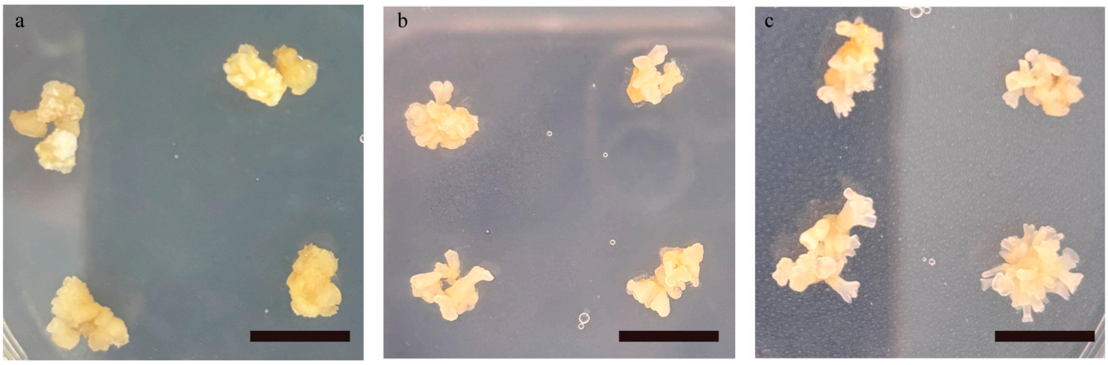

Figure S1 Representative pictures of peanut callus on induction medium after 2 (a), 5 (b), and 11 (c) subcultures. Bar = 2 cm.

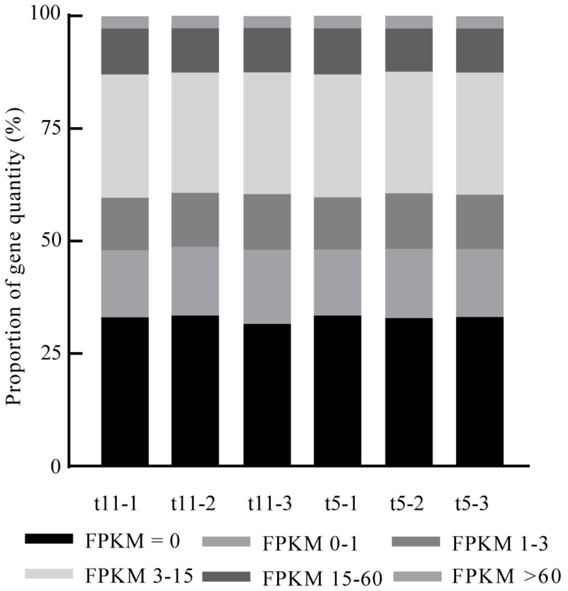

Figure S2 Stacked bar chart showing the distribution of gene expression levels in t5 and t11 peanut callus samples. Genes are categorized based on FPKM (Fragments Per Kilobase per Million bases) values into six expression ranges: 0, 0-1, 1-3, 3-15, 15-60 and >60. Each bar represents the proportion of genes falling within these ranges for individual biological replicates.

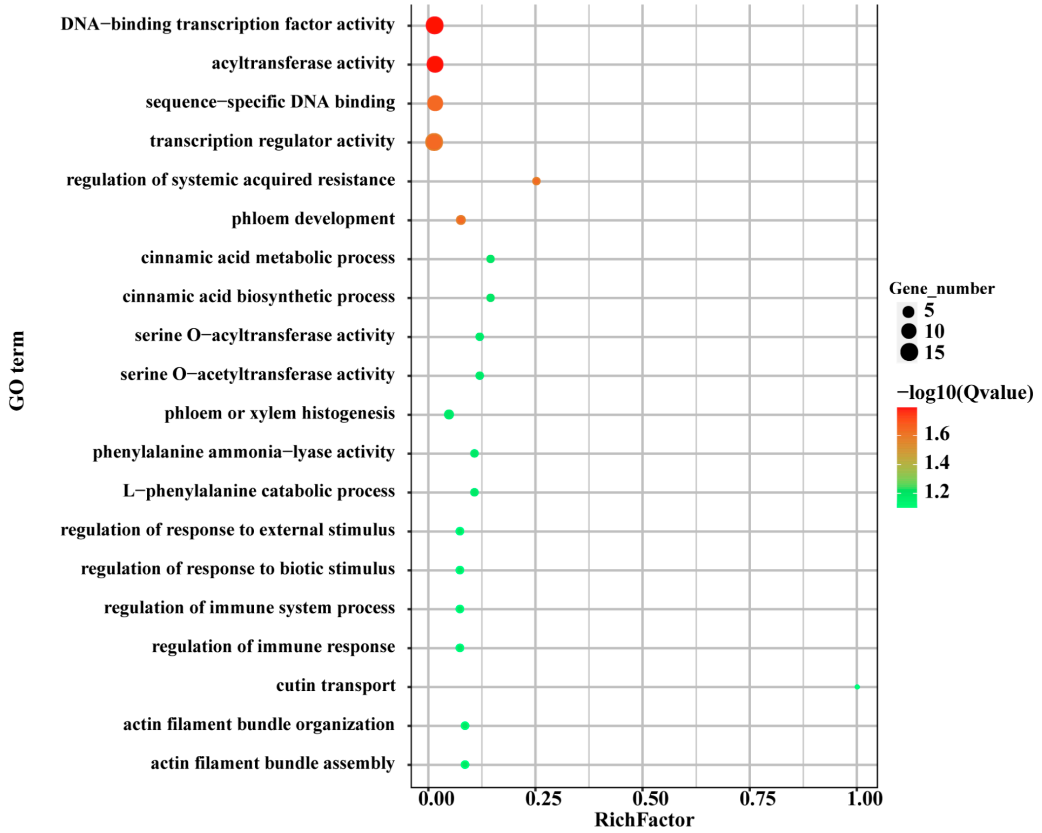

Figure S3 Gene Ontology (GO) enrichment analysis of the differentially expressed genes (DEGs) between t5 and t11 peanut callus samples. GO terms are categorized by biological processes and molecular functions. The x-axis represents the Rich Factor (ratio of DEG count to total gene count in a GO term), while the size of each dot indicates the number of DEGs associated with that term. The color gradient represents the statistical significance of enrichment, shown as $-\log_{10}$ (Q-value).

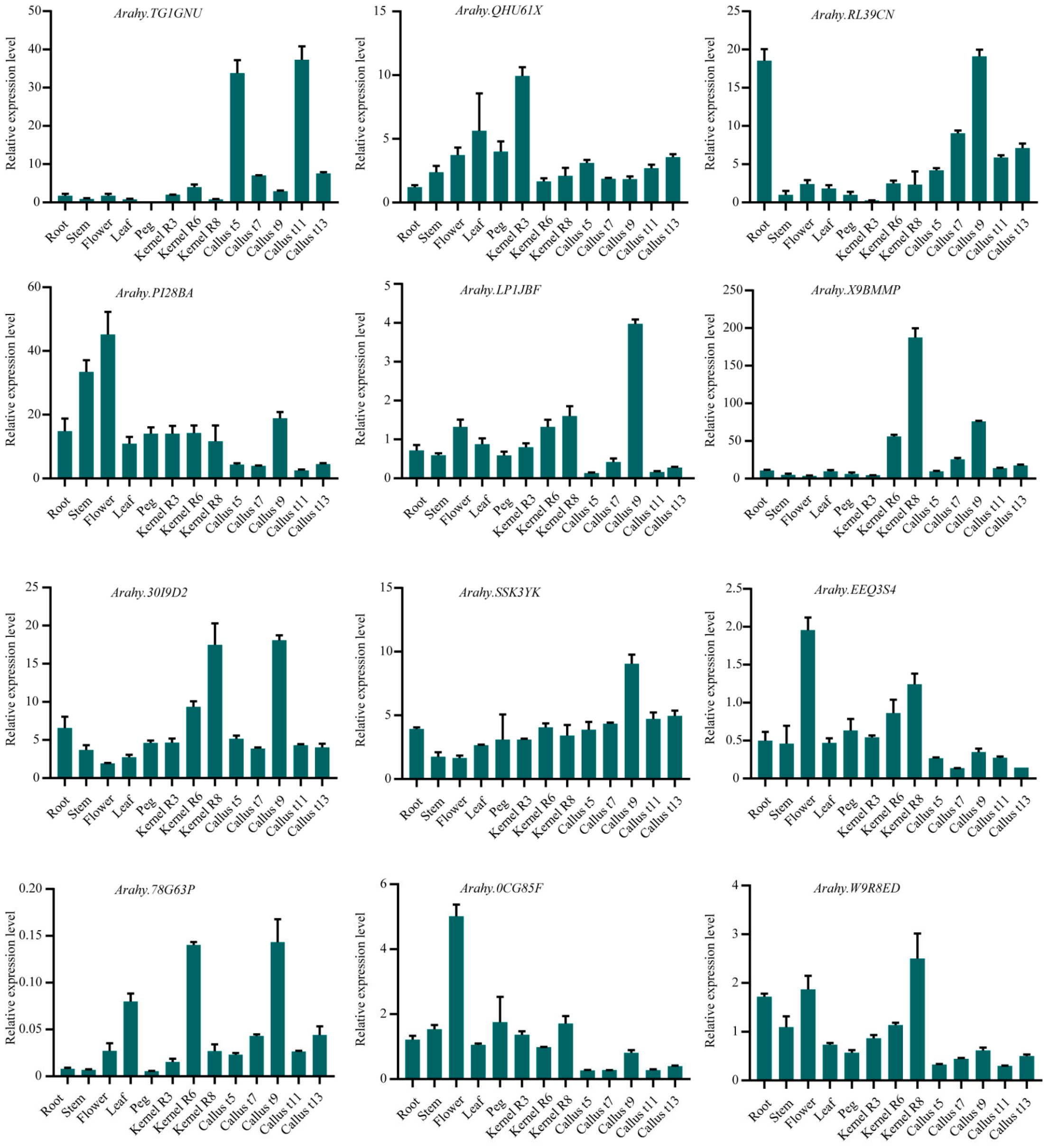

Figure S4 Relative expression levels of peanut callus-specific candidate genes across various tissues. Expression was analyzed in roots, stem, flower, leaf, peg, and developing kernel tissues at reproductive stages R3 (beginning pod), R6 (full seed), and R8 (harvest maturity), as well as in t5, t7, t9, t11, and t13 callus tissues. The housekeeping gene *AhADH3* was used as an internal reference. Data represent the mean of three biological replicates, each with four technical replicates.

```
-1723                    GTTTGGTTGGTAGCTCGTTGTGTGTCTTCTTCTTTATTTTATG
-1680 CTCCACTTTAATGTGTTGAGCTTTCTATGTTTCAAAAAAAAAAAAAAAACGAAAGAGCTGG
-1620 AATTTATTAGAAAGTCGTGACTTATTCGATGGTTATGACTTATTAAAAAATATTTTTTTA
-1560 AAGGGATGTTTTTTTTTCTTTAATGTATAAACTTAACGTCTATGAATAAAATGTTGGACT
-1500 GACAAAATAAAATACATTAATTTATATAATAATATATTTTTTTATTTTGTTATTTTTATC
                    Box-4
-1440 TTCTATTCAGTACTTTTTTTTTGTACATAAAAAAAAATTTATTTTGGATGTAAGACTTGA
                                                           W-box
-1380 CCGTGCGAATATAAAAGTAAATTAAGTGTTTTTTTTATATCCTACCAAAAAAAAATGATCA
         TATA-box
-1320 TTAGCTCACTTTATACACTATAATAGAAAATTAAATCTAAAAGGAATATGTAATATATTT
-1260 TTAAAAAATTATATCCTTATATTCTCTAATTTTTTTTATTGCATATTATATTTTACCTTAC
                         TC-rich repeats
-1200 TCATTTCTAAAGATTTAAATAAATTCTAAATATCCCTATCTAAAATTCTCACATTTTTCT
-1140 TCTTTAATAAGAAAATATAAACAACAATTAACTTAACCTGTGAATATTATCAGAAGTTGT
                 TATA-box
-1080 GAGATTCTAAATTTCTAATTGAAATTAAAATTATGAGTCTCTTTTAGAAGGGAAAAAATA
-1020 TCTTTACAACAATCAGGAAAAATTGGAAGGAGGCATAGAATATTAAATTTACCAAAAGTA
 -960 GCTACTTTTAGTTTGGTAAAGCTATTTTTAGTTTGGTAAAACTTTTACTTTTTAAAAGTA
 -900 GTTTATAAAAGTTAACTTTTAAAAGATGGCTTTTTAAAAGTTGTAGCATTTATGTTTGGT
 -840 AAATCAAATCAAAAATAACTTTTAATAAAGATAAGTAACATCAATTGTGTTTGGTAAAAT
        CAAT-box
 -780 AGCTTTTAAAATTTAAAAATACTATAATAGACATAAATGCAAACATTAAATTTGAAAATT
 -720 AGTTAACATATGAGGTTATATTAGACTTTTAAATTTTGAAAAGCACAAGACAACTTTGAA
 -660 AAGCTCTATCCTAGGTGCTTTCAAAAGTACCCCGATTTTTTAAAAGCTGCAAGTACAAGC
 -600 AGATGATCTTTTTGATTTACCAAACACAAAATGAGGAGCTTGAGCTTTTAAAAAGCACAA
 -540 ACACATCTTCAAAAAGTTTTACCAAACCAAGCCTTAATCATTCACCCTCATAAATCAGGA
                             ARE
 -480 CACGTGTCAATGGAGACAAAAAAGAAAAAGCATTGATTAGAGTATTTGAGATTATCTTAT
       ABRE
 -420 CCTTGTCCATTTTTGTAAAATTCAAAGTATGATACGATTCTCACTATCACTATATATGAA
 -360 TAGAAAATGTGTAGAACCATCAAAGTATGGCCCACAAGAATGGACCACAGCTTAAAAGAA
 -300 AAGTGGTAATAGCAGAAGCCAATAGATAGAATCATAGAATATACCAAACAAAATTTGAGC
         CTAG-motif
 -240 AATTGAACATAGCAAATTGGCATATCCCTTATTATATTAATTCATGTCACTTTTCTGTGT
              CAAT-box
 -180 GCCACCACATTCAACGTGCCATTAAAAAGCTCATAGATTATGCATGACACATTCATCATC
                ABRE
 -120 ACCCTCCTTTTATTACTTCCTATATAAACCAAACTAGCCTTAACCTTAAGACTTCATTCA
                          TATA-box                          ↑
  -60 GAAGCAAAAAGCACAAAAAAGTAAAAGCTAAAGGCTCTTATTGTTTTATCATCTAAGAAA
      ATG
```

Figure S5 Cis-regulatory element analysis of the DNA sequence of $P_{Ah\text{-}H0FE8D}$. The transcription initiation site is indicated by an arrow, and the start codon is highlighted with a box.

-2454 CACCGTATCCAACAAATTCTCAAAAAGAACCTAAACAGATATTATTTACTATAA

MRE

-2400 TATAAGAATCAATTCTTTGAATCCAAAAAATGACAGAAAATATTCCATCTTATTCTTATT

TCA-element

-2340 CTTAGTTGTTATGTTTAAAATAAATTATTATTAATTATTAAAATAAAATATTATCAAAAT

Box-4

-2280 ACTTTATATAATTAAAACAACATTAAAACAGTGAACAAAGTTAGTTTGTGCATTGTTTAA

-2220 TAATTTCTTTATATTTTATATATGTGTTTTCTTTTGTCTAACAAAAATTTAAAATTAGTG

-2160 AGTTTGTGTTAGTTACATTCGTGTTAATGTTAGCGTTTGTATAACATAACTTGAGAGGAA

-2100 AAGAAAACTTTTTAAAAGAAATCAAAACAATTATATTGATTGTTGAAAAATTACGAATAA

AT-rich element

-2040 ATAAAGTGCCTTAATTAACTTAACCTGCATCAGTCCCTTGTACTATATATCTAGATGCAT

-1980 ATTAATTATTATGATATATTGACTCGATCCCAATGATTCTCATGCTTTAATTTCGTTCCT

Box-4

-1920 TTGATTAGTACTATTTTTAGTTTCTATAATTTTTTTTCGTTTAGTTCAATTCTTTTTGTT

-1860 GTTGGGATGAGGAGTGCCCATGGTTTTCTAGCCTTCTCTCTGGACTAAACCGCTGGCTTC

-1800 TCCTACTGTTGCCATTGCGGATTAAAGAAAAATGTGTACTTTCTTTAACTTGCTAAATTG

-1740 ATATCAAGAAAACAAAGGATGTGTGTGAGGTTCAATGGAAGAAAATAAAGGGATCTTTTT

-1680 TATATGACTTTTGTGATGTTTGAGACACGAGAGGATAATGTCAGTATATATGTTTAATGT

-1620 TAGTATTTGTTATATATATATATATATATATATATATATATAATTATTAAAAATGAATCC

-1560 TCTTAATTTTTTTTTAATAATTGAAAGAGTAAAGTGTGATTATTTACTATTAATTTTATA

Box-4

-1500 AATAAAATCAAGAATAAATATGAGAGAAAAAATAATGAAGAGTTAAAGATACACTTTATT

-1440 AATTTATATATATAATTAGATATTAATTTAGTTAAATATATCAAATCATCTAACCATCCT

Box-4

-1380 TAAATATAAACTTCATATTATATGGATTTTTAACTTCTTTTTAATTTATTATTAGATCAA

TATA-box

-1320 AGTTTTAGCATCTCCAACAATTTTATAGTTAAATGAAAAAATTCTAATTAATTAAATAAT

-1260 ATAGATTTTTAATTTTTTTCTTAAAAAAAGAGAATAATAAAATTATGAATTATATAAAAA

TATA-box

-1200 ATACTGATATCAACATAAACTTTGTTAGATTGCATTAGTTTTAGAACGAGTAATCTTACA

ATC-motif

-1140 TATTCAGAAAATATTATTATTTTTTATTAGTATTTAGTTAATAATAATTTATACTAATAT

AT1-motif

-1080 TTATAGGAGTTTTATACACAAAAAACATATAGTTTATATCTATATTTATTAGAGTTTTAT

-1020 ACACATAAATTAACACAATTTATACCTATACTTTTTTAGAATTTATACACCTAAATTAAT

Box-4

-960 AAAATTTATCTGTTAAAAATAATTTAATATTTATAATGGTCAAATAATGACAAAAAATAT

CAAT-box

-900 TAAAACTTGTTAGTCTAAAAAAGATTCTCTTTTAAAATTTTATTTTAGTAGAAAAGTTTT

-840 TGAAATTTTTATTTTAATAAATAAAAAATAAAAGTATTAAAAACTTCAATTTTACATATT

-780 TCCATAAAAGAAATTTCAATTAAAGCACGTGTTAACCAGACATTTGATTTTATTCGTTTA

ABRE

-720 AACAAGATTAATTTGAATTACAACTTTATAAAGTAGATGATAATGCATTAATTTTTGGGA

Box-4 TATA-box Box-4

-660 AGACAAAAGAAATGAAGGGACAAGGACAAGGACTTAAATGAAAAACCACTGCCTGAGACG

GATA-motif ARE

-600 GTGGATTTAGGGAGAAACCATCAAACTTCACCTGCCAAGGACGGGTGCAATGCATAATGC

ARE

-540 ATGCACGATGCAACTCCCTCTCTAATTGTGTAATAAAAATAGATAAAGTAGTTAAATCAT

GA-motif

-480 AAAATATGTGTTAATATTTTATTTTTTATATTGTCTACTAAGTTATTAATGTATATTTTT

Box-4

-420 CTCTAATTAAAAAGAGTGTGATATTATGTAAAATGATTTATTGTAAGAAATAAAATATAT

-360 ATTATTTAGAGAAAAAACTCAAATGTAGTTATTTTTATGTGAAGTTGTTAATTAAAAATT

-300 GTTAAATAATTTAATATATTTGACTAAATTATAATATAACGTTTCTCAACTAACAATTTT

CAAT-box

-240 AACTACATGTGAATTTTTAACGTATTATTTATTAAAACAAGAAATATATTAGTAAATGAT

ACE TATA-box

-180 GGTCTAGGGAGCAGCTCAGTTATAAATATGGCAGCAACTTAAACCCTCTCTTCACTCAAA

↑

-120 TTAAAGCTATTGAGTTATATATATTGTATTGCTCTGCTTCCTAAAATTGAACTTCTATTA

-60 TTCTTCCATTAATGTTATACATTTTTTCTTGAATAAAAGTAAGATATATAATATCTGAAT

ATG

Figure S6 Cis-regulatory element analysis of the DNA sequence of $P_{Ah\text{-}WT3AEF.}$ The transcription initiation site is indicated by an arrow, and the start codon is highlighted with a box.

```
-2116                                                    CAAGCGAATAATCCTA
-2100  AAAGGCGCAAATTATAGATAAAAGCTGAAATGAGATTGGAAACAAATTCAAGAAATTGAG
                    GA-motif
-2040  AGCAGAATAATTGAAGTACCGGGAGGAGAGGAGGTTATCGGCGACGAAGGCGGAGGAGGA
-1980  GCAACGGCAGGGTTCGAAGAAGTGCTTTGGAGATTTGTGGAAGCGTTGGTAGAAGAGGCG
-1920  CGTGAAGAAGGTTTATTGAGGCGAGGTTTAGAAGAAGTAGAAGAAGGAGGTTTGGATTTG
-1860  GGTTTGGGTTTGATAAGGTTTGTGGAAATTGGAGAAGAAGAGTTAAGAGTGTTGGTGCGA
-1800  TCGGATAGAGGGTTTCTGCGTTTAGTGCTTCGGTCAGCTTCCATCTTTAGATCTTCGGGA
-1740  ACTCGGTTCCCATTTCAATTGAAGAAGAAGAAGAAGAAGTAGTGATGCGATGATTGAAAA
                WUN-motif
-1680  TGGAAGATTGAAGAAGCTGCTTGTTTGTTCGTTGAGTGTGTAGTGTTTTCTCGTCTTTCC
-1620  TGAATAGCGGGAATTTTCAGTTGAACAAGAAGGATTAAAAATCCTGAGGCAAGCTTCCAG
-1560  AGGAAGACTCCAATAGTCCACTGATTATAGTCAATGACAACGAACAGAAACCTCAGCTGC
               CAAT-box             CAAT-box
-1500  TTTTTTTTTTTTGGTAATATAGTGTCTTTTTGGACATGAATAAAGTTTATATGGGCCAC
                                                           CAT-motif
-1440  TGATATAACACTCATACAAGCTGGGCCGATTCACATGTTTTAATACATGTGAATAGGCCC
-1380  AAAATAGGCCCATGGAAGAAAAACCAAATTGCTTTTTTTTTTCTAAGATATTATATTTCA
                            ARE
-1320  TTGATAAAATGAGGACATTAGGACTGAGTTGAAAATTTTATTAAACTCTATCTTATCCTA
-1260  TTTGTGGGTGGAGAATTTCTCAACCCTAACCCTACCCGCATTCTAAAATTCTAAACCCTA
-1200  ACATATCCTATTCTATCCGCAAAAAATATCAAATTTTTTTAAAGTAAATATAAAATTCAA
                                                      TATA-box
-1140  TCATTTTGAATTTTATACATATTAATAACATAAAAAATAAAAAATTAATGTTCTAAATTA
                           Box-4                     WUN-motif
-1080  CTAAATTAACTAACTAGTTTTAATGGTTGTTTACTTATTGTAAGTTATTACATAAAAAAC
                                                   WUN-motif
-1020  GTTATGGGTTCAACTCTCATTTCCTTCATTATAGGGTAGAGTAGGGTGCGGATTTTAGAT
 -960  GAGATTCTAGCTCAATATAGTGCCTAATATTTATTTATAAAATTTAAAAATAAAGGGATG
                                         TATA-box
 -900  AGTCTCACTTTATTCTATCTCTATTTTTAGATAGGTTAGACAAAAAAAATTTATAAGTAC
 -840  CAAAAAATCTACTTTATTTTATATTATTCATTGTTTGACATGTCCGTAATACAAATTTAT
 -780  TAATCTTTTAGATCATGAGTAATTTTTTATAGCTGCTTTAAAAATTTAACTTAAAATAAA
       Box-4
 -720  AAGAATACAATTTTATTGCTCACATCTTAAATAATGAATGACAGAGAACAAACGGCTTGA
            CAAT-box
 -660  TTTTTCCTTGAAAATTTTGTCTTTTTTATTTTTTATTTTTTTAATTTCTCTACTACCGGA
 -600  AATGTGGAGATTAGTTGTATTTTGAGTATGAAAAATGTAGTAAAATTATCCCTTCTTGAG
 -540  ATTGATAACCGTTTTCAAATCTCAACCTGTACTTGGTGGTAGTTTAATGAATCTAATGCA
                     CAAT-box                           ATCT-motif
 -480  TGGACGGTGCCTACTCAGAAAAAAAGGTATATGATTATAAATAATTAAATATAACCCACA
                                         TATA-box
 -420  GGTCACAGATATAAATGGTGCTCATTTTTCCTTAACTTGATGCAGCACACAGTCACAGCC
               TATA-box
 -360  TCAAAACTCAAAAGCCACTTTGTTTTTTATCAGTTTTTATGTTAAAAAGCTATTTTCACA
                  CAT-motif
 -300  TGGAAACATAAGAAAATCATAAGGCACGTGCATAAAAATCAGTTACTCAAAGTAATTTAG
                                ABRE          ↑
 -240  TTGAGACTGAGAAGAGCAGAGACAAAACCGGGTTAAGCCTCTAGTTTAACCACTTTTCTG
                                   GT1-motif
 -180  TGTTCCAACAAACTAAAGGTGTGACCCAACACACTAAAGTCTAAACCCGCTAACACAAAC
 -120  AAACCCAGGGGCCACATTTATATAACATTTCAATTAATTAATAATTTAATTTCACCTACT
             STRE
  -60  ATATAAGTAGTATATCTACTACTCTTTCCTTTCACAATAACACTATACAACACAAATAAC
       [ATG]
```

Figure S7 Cis-regulatory element analysis of the DNA sequence of $P_{Ah\text{-}I20Q6X.}$ The transcription initiation site is indicated by an arrow, and the start codon is highlighted with a box.

```
-1938                                                   TGTGGTTTGTTTGGGACT
-1920 CTTTTATACCTCATTTGATTATAATGGAACTATTTTATTGGTCTTGGCGATTCGTGGTTT
-1860 TTACCTCTCACATTGAGAGAGGTTTTCTACGTTAAAATCTCGATGTATTCTTGTTATAGC
-1800 TTTACTTGCTATATTTGCTTGTTCATAGTACTGTCATATTGTGTTTAAGAGTATTTTCAT
-1740 ATTATTCTTGTGTTGGATATTTGTGTTATTTTTATTGCTAAGCTCTTTCACAATTCAATA
-1680 TATATATATATAAAATACGTATTTTCGGATATTGTACAAAGATTATTATTAATAAATATA
               TATA                                          Box-4
-1620 TTAAAAGCTTAAATTTGAATAATCTTTATAAAAAAATTAATTTGCAATCTTAATATATAT
                                              Box-4
-1560 TTTAACACATATTAGTTAAGTATTTATTTATTTATTGAGCATAGCAATCATTGTTAATTG
                                                     HD-Zip 1
-1500 TTAATGTTGGTTAATGGCATTTGTTAAGCCCCCTATGAACACATTGCTAGAATTAAAATG
             GT1-motif
-1440 CAGCCCATGGCTTTGTAAGAAAACAATTGGCATTTGTAATCGACAACCTTGAACAACCTT
-1380 TATTACTTAGAAATAACCAACTCTTTTGTCAAAAACCATGTGCTCTCAAAAATTCCAATC
                                          ARE
-1320 ACTCTTTTTCAAACAAGACCCTTCATTCATGCAATTTCAATATGCGATCTTTGAATTGAT
-1260 TCAAGGGGATGTAGTGGACTACAAATCAAAGTTTAGATCATTGGATACGCCAGAATAACA
        STRE             CAAT-box
-1200 TTTAAAAATATAAATTTTTAAAAGAAATAATGTATAATACGATATAAAAAATTTTATAAA
-1140 TAAAAAAAAATTTATTTAACATTTTTGTCTTTTAGAAGATATACTTCTTGTTCTTGATCT
-1080 ATAATATTTAAGAAGAATTTTAAAGGTACCAAATACACCGTTAATTCAATGATTTTAATG
                        ERE          CAAT-box
-1020 GTTGATTTTAATTATAAAAATATATATAATATATATTATTTAAAATGAATGGTTAAGATT
                TATA-box                                   GT1-motif
 -960 ACTAGAATACTAGTGTTTTGGATGCATTTGAAAACTTTCTTAATATTTATACATGATTAT
 -900 CATGGAATGGATGTGAATGTGTAATTATTTTAATTTTGATACACTGATAGTGTAAAATAT
 -840 TTTATACCTTCATACAATTACGTTTTTTAATTTTATTATGAAGAAGGTAATGGTGTAAGA
 -780 CAACAATATGGAGAAGCTAGTGCTTTACAGAGGCATATGGTGTCAAAGAGTTACTAATGG
 -720 GACAGTGCGAATGCTCCCAAACAAATCGAACAGTCCGATTTCAAAAAAAAATTATAATAA
 -660 TCAATTCGGACCATTCAATTTGATTTTCGTTTTATTAAAAAAAAATTTCATACAACACAGA
 -600 CAGTTCAATTTGCTTTTTTCAAAATTTAAAATTTTTCTCCTACAAATCACACCATCCGAT
 -540 TTGTCTATAATATTTTTTTTTTAAACATAATTCGCATGTTCCGACTTGTCTATAGCACAA
 -480 ATAAAAAAAAGATTACCCCCACAAATTAGTTTAATACCACTATATTCCATAAAACAGCAC
                           CAAT-box
 -420 ATATATGCCTCCAATAACAAAAGATGAGCCCATGCAATTACAACAATTTTTTTAAATGAT
 -360 GAATCAATATAAATGAATCAATATAAAAATAATTATTTTTACTAATACGAATGCATACGC
                           TATA-box
 -300 TAATAAAACAATTTTACACCCTTTAAAATATAAAAACTTATATTTAAGATAAGGAGCACA
                                                        I-box
 -240 AGAGTCTCATGAAAGCCTCAACTAAATATCAAATCTCAGCCATCCATAATATCTTTCTCT
 -180 TTTCCACCAGCAACGGTCGACATTCAATGTCCCTTCCAATTCCGCCACGTGTCAAGCCCC
            CCAAT-motif               CAAT-box    ABRE
 -120 TCTATATATATACCTCGCCTCTCCCTCCACTTTCTTTTCACTGTCATTTTCTCGGGAAA
       TATA-box       TCCC-motif        ↑
  -60 ACTGAGGGAGTGAGAAAACCGTGAAAATTTTGAAAAACCACTGCGATTCACTGAACAACG
      ATG
```

Figure S8 Cis-regulatory element analysis of the DNA sequence of $P_{Ah\text{-}ELJ55T.}$ The transcription initiation site is indicated by an arrow, and the start codon is highlighted with a box.

```
-2116                                                   TTAAAGCTCTTACTTC
                                                               TCT-motif
-2100  TTTAGGAGTTTAAAAGATGATTCTTAAAACAAGAGAATTAAATAAACAATCTTATACTTT

-2040  CATAGCTATGTGACGAAACTTAAGAGGAAAAAGAGAGAAAAAAATTCTCTCACCTTTTAA
                 as-1                               TC-rich repeats
-1980  GTTTAAAATTTGTGATTTTCTACTCTTTGAGGATTAAGAACATGATTCCTAAACAAGAAA

-1920  AGATAAAAAAATTAAAATAGATCATCCATGGCAATTTTCTCATGGCCGAATTTATGTGAA

-1860  GGAGAAAGAAGAGAATTTTTTTGACTAACCTTGATTAAATTGGTATGGATGAGAAGAAAA

-1800  TTAAGACAGAAATTAATTAATTGGAATAGAATTTGGATTACAGCCTAAAATCCTTCGTAA

-1740  ATCAAGCATGAATTTTAGATGAGCATATTTGGTTCTCTCTTTTCTCTCATATTTCTTATG

-1680  AAAATAAGGAAGAAAATAAATTATAATAAATGAGAGAAACAGAAAAAGTCTATGAAAATA

-1620  AAGATAAAAGGATAAGATTGTCGCTCAAAATATTTCTTAATTAAGAGATAATTATCAAGT
             GATA-motif
-1560  GTGGTAATTATTTTACATGATTTTATTTTATCTGGTCAATTAGTTCTTTTATTAGTGTCA

-1500  ATTATATTGATATGATAAATTATCTCAACTCTAAAATAACTCTAAAATATTTTGGTATAG

-1440  TTTACTAGAACAAAGAATATACTAATAAAATTCTCTTACTAGTTAAATATTAATCCGATA
                                        TCT-motif
-1380  ATTATGTAGTCTAAAAAAAATCTTTAATTATTCACAAATTAATATTATTTTACAAATAA
                                            Box 4          CAAT-box
-1320  TAATTTTAAATATTTTTATTTTTGATACGGGCTTCACTCATTAAATCGAATTAATTTGCG
          ERE                                             Box 4
-1260  ATACACGATTTTTTTAGATTCTGATCAtAGAAAATTCTAAAATACGAAATATTGGATTGA

-1200  TACTTCAACAATATGAAAAATTTATGAATTTGAAGTACTTTTCTTAAAGTACCCATTAAT
                                                                 Box 4
-1140  GTTAATTATAAATTATGAGAAAAAAATGGACATAAAATTATATTTGATTCTGAGAATAAA
          TATA-box
-1080  ATAAGATAAAACACTAAAAATAAAACATAAAGGATAGAGATACAAAAATTAGTGTTCTAG

-1020  GATTTTGTTTAGTAATAAACTAGAACAAATTATGAAAGTCTAATTTATTCTATTTTTTAT
                                 CAAT-box
 -960  TAAAAAATTTGAAAAAAATATAATTATAAAAAATTAATAAAAATAATAAAAAAAAATAAAA
                                 TATA-box
 -900  AATAAGTTGTCTCTGTGTGTTCTAATTGATAAGATAAACACAAAATATACTAATTTAATA

 -840  TTTTTAGACACAACATCTCTATTTATATCTCATCTATCAAATATTGTGTTCCTATGTCTA
                                             CAAT-box
 -780  TATCTTAGTGTCTTGTACCTATATACACGAACATAGCGCAACCTAATATACTTGTTGGTA
                                                  MRE
 -720  GTCACTAAAAAATTAATTTAGATTCTCTAAATTTTAGATTTTTATTTTAGAGGATAAAGT
                                TC-rich repeats
 -660  AAAATCTCTCACCAATTTTTTCTTTCATGTATTTTTTTGTCCCACCTATTAAATAAATAA

 -600  GATAGATCATACTTTATCTTCTAAAATTAAATTTAAAATTTAAAAAATTCAAATCCCTAA
                                                          CAAT-box
 -540  AAAATAAATTAAATTAAAGTTTGTAGACCGATTTTTTTATTCAGATCTTTCATTTTTAGA

 -480  AACTTATAAAGTTCTTCTCTTAAGCTCCAATCAATGAACCTACAAAACCCTTTAAATTGT
       AE-box
 -420  ATCTCTGATTCAATCCCAGTATTGTAGACATTCCCGCATTTTCACCCCAATCATTTTTTA
                                                        CAAT-box
 -360  TTTTCCCCTTGTATTTTTTAGAAAACCATTTATCTCCGCCTATTTTCATTATAAAGGCAT
                                ARE                      TATA-box
 -300  CCGTTGACAGCATTTATGTACTTTGTACCCTTCCATGATATGAAATTATGGATACATTAT

 -240  TTGAATTCTTTCTTGCATAAGATAATGTTGTTTCTGCATGCAATCAGCGAAACCCATGTT

 -180  CATACTTTCAACTTTGCTACCATTGATTTGAAAACAAGTTTCCAGAAGCCACCTTTTGCA
                               ↑                                 P-box
 -120  ATAACCAAGCAACATGTATAGACATATTACTTCTATAAATAACAGGCGAAACATTGAATT

  -60  GCTACATCAATTAGCTCTAATAATAATAACTCGACTTGATTTCTATACGAGAATTTAAGT

       ATG
```

Figure S9 Cis-regulatory element analysis of the DNA sequence of $P_{Ah\text{-}N9CMH4}$. The transcription initiation site is indicated by an arrow, and the start codon is highlighted with a box.

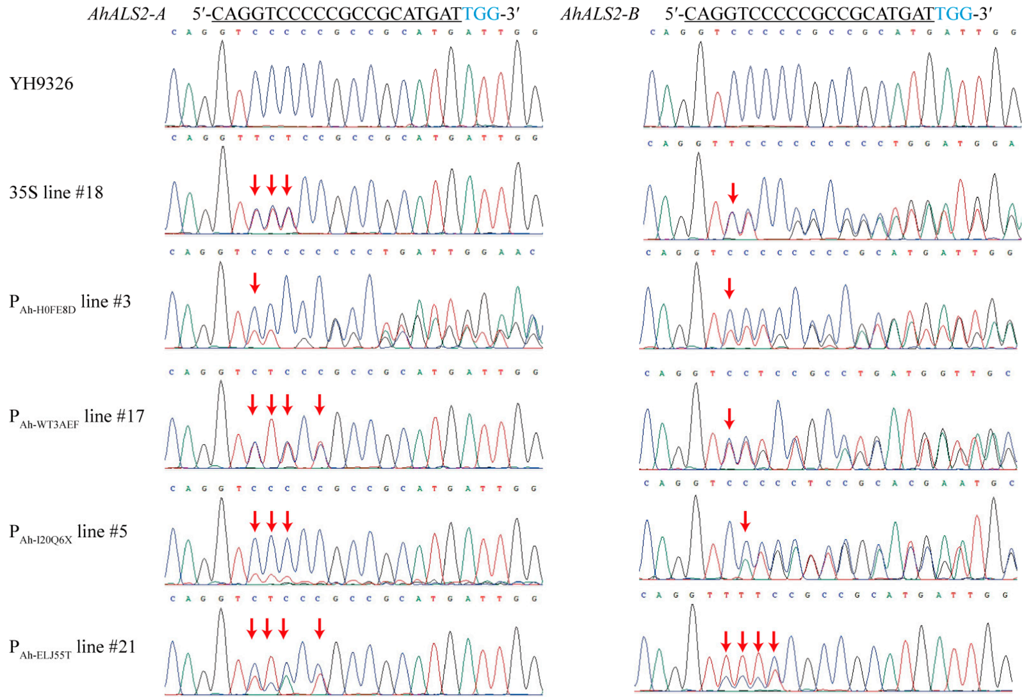

Figure S10 Representative Sanger sequencing chromatograms of the edited target sites of *AhALS2-A* and *AhALS2-B*. Target sequences are underlined, with the protospacer adjacent motif sequences highlighted in blue and nucleotide mutations highlighted in red.

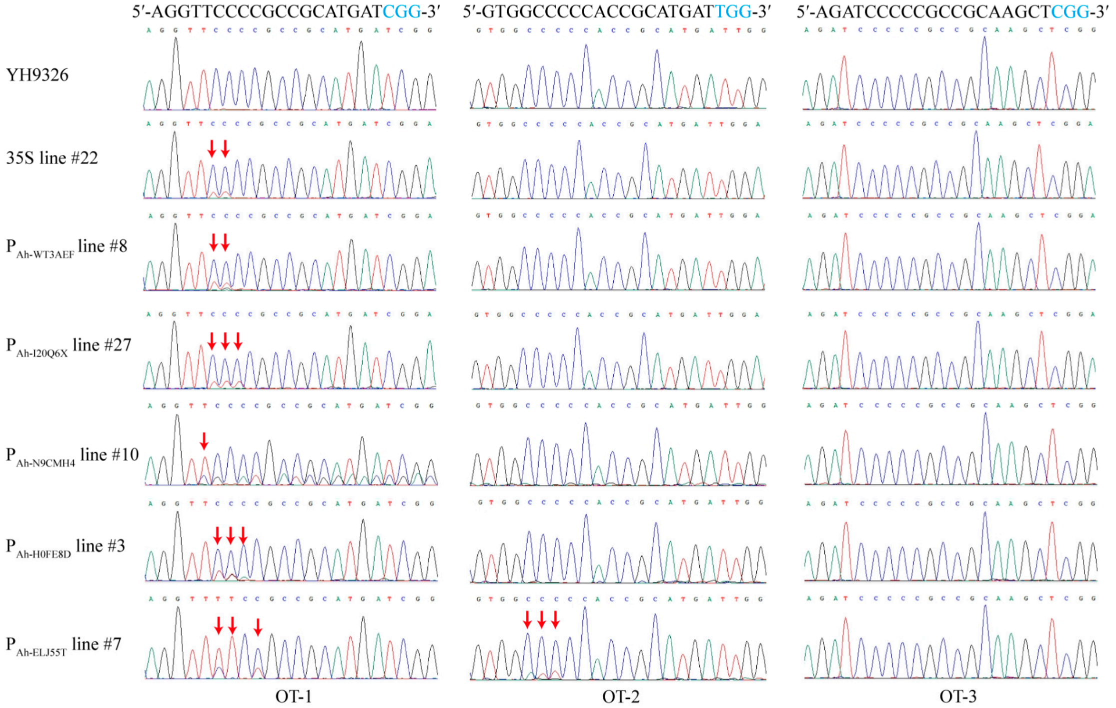

Figure S11 Representative Sanger sequencing chromatograms of the off-target sites in edited lines. The protospacer adjacent motif sequences and nucleotide mutations are highlighted in blue and red, respectively.

Table S2 Peanut callus-specific candidate genes

Gene name	Gene annotation
Arahy.TG1GNU	Uncharacterized protein DS421_17g569330
Arahy.H0FE8D	Uncharacterized protein DS421_3g64140
Arahy.WT3AEF	Early nodulin-93
Arahy.I20Q6X	Uncharacterized protein LOC107496957
Arahy.QHU61X	Histone H2A
Arahy.ELJ55T	Enolase
Arahy.RL39CN	Uncharacterized protein LOC112697982
Arahy.PI28BA	Heat shock cognate 70 kDa protein 2-like
Arahy.LP1JBF	Glycine-rich protein 2
Arahy.X9BMMP	Transcription factor KUA1-like
Arahy.30I9D2	Uncharacterized protein LOC107482080
Arahy.SSK3YK	Hypothetical protein Ahy_A02g005636 isoform E
Arahy.KXQV93	Early nodulin-93
Arahy.EEQ3S4	NADH dehydrogenase [ubiquinone] iron-sulfur protein 5-B
Arahy.78G63P	Developmentally-regulated G-protein 3-like; 60S ribosomal protein L6-1
Arahy.0CG85F	Uncharacterized protein LOC107466559
Arahy.N9CMH4	Uncharacterized protein LOC112790019
Arahy.W9R8ED	Hypothetical protein Ahy_A07g031648; small ubiquitin-related modifier 2

Table S3 Off-target site prediction of P197 target site

Off-target site	Putative off-target sequences （5′-3′）	Nucleotide mismatch number	Off-score	Chromosome position
OT-1	AGGTtCCCCGCCGCATGATCGG	1	0.636	Arahy.08: 50532271..50532292<br>Arahy.18:134618612..134618633
OT-2	gtGgCCCCCaCCGCATGATTGG	4	0.371	Arahy.17:80268988..80269011
OT-3	AGaTCCCCCGCCGCAaGcTCGG	3	0.130	Arahy.12:94705742..94705762

Note: Lowercase letters indicate mismatched bases.

Table S4 Off-target analysis of peanut callus-specific promoters

Vector	Number of genotyped lines	Off-target rate		
		OT-1	OT-2	OT-3
35S-evoCBE	4	75.0%	0	0
$P_{Ah\text{-}H0FE8D}$-evoCBE	6	50.0%	0	0
$P_{Ah\text{-}WT3AEF}$-evoCBE	4	25.0%	0	0
$P_{Ah\text{-}I20Q6X}$-evoCBE	2	50.0%	0	0
$P_{Ah\text{-}ELJ55T}$-evoCBE	5	80.0%	20.0%	0
$P_{Ah\text{-}N9CMH4}$-evoCBE	8	37.5%	0	0

Table S5 Primers used in this study

Primer name	Primer sequence (5′-3′)	Application
q-cal50-f	GAGTATCCTTCGTCGCCTCCTTG	Amplifying *Arahy.TG1GNU* for RT-qPCR
q-cal50-r	CCAGACAGCACTCAGGACCCAG	
q-cal104-f	CTGGCTAGTGTAAGGATGGTGC	Amplifying *Arahy.WT3AEF* for RT-qPCR
q-cal104-r	GAGGGTTGTTGGTTGAAGGAGT	
q-cal142-f	GTTACTTGAAGAAAGGAAGGTACTCC	Amplifying *Arahy.QHU61X* for RT-qPCR
q-cal142-r	AGAACATGCCTTGGAATAATCCTG	
q-cal55-f	TGCTATGGTTGATGTATGGGTT	Amplifying *Arahy.H0FE8D* for RT-qPCR
q-cal55-r	TTCCACCACTTTTGATGAACTT	
q-cal165-f	GAGGAACCGTTCCCTTACCAAA	Amplifying *Arahy.RL39CN* for RT-qPCR
q-cal165-r	ATGACTCATTTCCACGGCCTAA	
q-cal226-f	GGTCGAAACAGAAGGCAATAAC	Amplifying *Arahy.X9BMMP* for RT-qPCR
q-cal226-r	TAGGATACAGCACTGGGAATGG	
q-cal253-f	ATGGGAAGAAGTGAACAGGATG	Amplifying *Arahy.SSK3YK* for RT-qPCR
q-cal253-r	ATGTGGTGGGACAGAATAATGG	
q-cal279-f	CAAAGGGAAATGTTACTGCGAATTA	Amplifying *Arahy.KXQV93* for RT-qPCR
q-cal279-r	CATCACTCCCTCACGAGAACAAC	
q-cal330-f	AGAACTCGGTCGAAGAGGGTAT	Amplifying *Arahy.0CG85F* for RT-qPCR
q-cal330-r	GCTTTGCGTGCATGTAGTATGG	
q-cal339-f	ATGTGGTGCCAATCACTAAGGAAGAC	Amplifying *Arahy.N9CMH4* for RT-qPCR
q-cal339-r	GCATTGCGACACCTGGCTTC	
q-cal136-f	TCACTGATTGCTTATTCAAGAGCAAG	Amplifying *Arahy.I20Q6X* for RT-qPCR
q-cal136-r	TGATATTTGGTTCATTATTATTAGCCAT	
q-cal168-f	AGAGTTAGCGATGCCTCAGTTC	Amplifying *Arahy.PI28BA* for RT-qPCR
q-cal168-r	TTGCTTCTCCTCACCCTTGTAG	
q-cal144-f	TGCTGCAGTGAAGAAAATTCCG	Amplifying *Arahy.ELJ55T* for RT-qPCR
q-cal144-r	CTGCATTGCCAGCTTATTTCCC	
q-cal233-f	TAGAAGCTGCCCTTGCCGCT	Amplifying *Arahy.30I9D2* for RT-qPCR
q-cal233-r	TTGCTCTTGGCAATCTTCAATTTC	
q-cal316-f	ACGTATCGTTGAGCTAAAATTCAGG	Amplifying *Arahy.78G63P* for RT-qPCR
q-cal316-r	TATTTACTATGGCTCCGCAACCTAG	
q-cal341-f	TACGTAATCTAGTGTAGTTGTTAATTTGG	Amplifying *Arahy.W9R8ED* for RT-qPCR
q-cal341-r	AGAGGAGGCTCTTCTAAGACATTATAG	
q-cal204-f	AGCCTCGCCGAGGGTGAG	Amplifying *Arahy.LP1JBF* for RT-qPCR
q-cal204-r	CACCACCATATCCTCCGCCT	
q-cal313-f	CATCATTAGCACTCAATCAGTTATCAG	Amplifying *Arahy.EEQ3S4* for RT-qPCR

q-cal313-r	CAATGTTGAAGGGATAAAAAGAAGG	
Pcal136-f	CAAGCGAATAATCCTAAAAGGC	Amplifying promoter of *Arahy.I20Q6X*
Pcal136-r	GTTATTTGTGTTGTATAGTGTTATTGTG	
Pcal339-f	TTAAAGCTCTTACTTCTTTAGGAG	Amplifying promoter of *Arahy.N9CMH4*
Pcal339-r	ACTTAAATTCTCGTATAGAAATCAAG	
Pcal144-f	TGTGGTTTGTTTGGGACTCTTT	Amplifying promoter of *Arahy.ELJ55T*
Pcal44-r	CGTTGTTCAGTGAATCGCAGTGG	
Pcal55-f	GTTTGGTTGGTAGCTCGTTGTG	Amplifying promoter of *Arahy.H0FE8D*
Pcal55-r	TTTCTTAGATGATAAAACAATAAGAGCCT	
Pcal104-f	CACCGTATCCAACAAATTCTC	Amplifying promoter of *Arahy.WT3AEF*
Pcal104-r	ATTCAGATATTATATATCTTACTTTTATTC	
SG-P197-F	attgAGGTCCCCCGCCGCATGAT	Annealing for production of double stranded P197 sgRNA
SG-P197-R	aaacATCATGCGGCGGGGGACCT	
15-bp-gus-F	ccaatacgcaaaccgcctgcagg	To add to promoter amplification primers
15-bp-gus-R	ttaccctcagatctaccatgg	
15-bp-cbe-F	ccaatacgcaaaccgcctgcagg	To add to promoter amplification primers
15-bp-cbe-R	ttccgcttcttctttggtaccatggtggc	
HT-197F	GGAGTGAGTACGGTGTGCGTCTGCATCGCCACCTCC	Amplifying P197 target site of *AhALS2A* and *AhALS2B*
HT-197R	GAGTTGGATGCTGGATGGAAAAGCCTCATTCACAATCCTA	
197OT1-F	AAGGCGTAACCACCGTCTTC	Amplifying P197 off-target site OT-1
197OT1-R	CCTCCGAAACTAGCCTCACAAC	
197OT2-F	TTAGGATTTGACTAGGACGTGTAGAT	Amplifying P197 off-target site OT-2
197OT2-R	CTTTACCTCTTTCTCGCTTGCTT	
197OT3-F	TTCTGCAAGACCTGTAGAAGGTAC	Amplifying P197 off-target site OT-3
197OT3-R	CTCCCATTGATCCGGAAATC	

Gene sequence

$P_{Ah\text{-}H0FE8D}$

GTTTGGTTGGTAGCTCGTTGTGTGTCTTCTTCTTTATTTTATGCTCCACTTTAATGTGTTGAGC
TTTCTATGTTTCAAAAAAAAAAAAAAAACGAAAGAGCTGGAATTTATTAGAAAGTCGTGACT
TATTCGATGGTTATGACTTATTAAAAAATATTTTTTTAAAGGGATGTTTTTTTTCTTTAATGTA
TAAACTTAACGTCTATGAATAAAATGTTGGACTGACAAAATAAAATACATTAATTTATATAAT
AATATATTTTTTTATTTTGTTATTTTTATCTTCTATTCAGTACTTTTTTTTTGTACATAAAAAA
AATTTATTTTGGATGTAAGACTTGACCGTGCGAATATAAAAGTAAATTAAGTGTTTTTTTTAT
ATCCTACCAAAAAAAATGATCATTAGCTCACTTTATACACTATAATAGAAAATTAAATCTAAA
AGGAATATGTAATATATTTTTAAAAAATTATATCCTTATATTCTCTAATTTTTTTATTGCATATTA
TATTTTACCTTACTCATTTCTAAAGATTTAAATAAATTCTAAATATCCCTATCTAAAATTCTCAC
ATTTTTCTTCTTTAATAAGAAAATATAAACAACAATTAACTTAACCTGTGAATATTATCAGAA

GTTGTGAGATTCTAAATTTCTAATTGAAATTAAAATTATGAGTCTCTTTTAGAAGGGAAAAA
ATATCTTTACAACAATCAGGAAAAATTGGAAGGAGGCATAGAATATTAAATTTACCAAAAGT
AGCTACTTTTAGTTTGGTAAAGCTATTTTTAGTTTGGTAAAACTTTTACTTTTTAAAAGTAGT
TTATAAAAGTTAACTTTTAAAAGATGGCTTTTTAAAAGTTGTAGCATTTATGTTTGGTAAATC
AAATCAAAAATAACTTTTAATAAAGATAAGTAACATCAATTGTGTTTGGTAAAATAGCTTTTA
AAATTTAAAAATACTATAATAGACATAAATGCAAACATTAAATTTGAAAATTAGTTAACATAT
GAGGTTATATTAGACTTTTAAATTTTGAAAAGCACAAGACAACTTTGAAAAGCTCTATCCTA
GGTGCTTTCAAAAGTACCCCGATTTTTTAAAAGCTGCAAGTACAAGCAGATGATCTTTTTGA
TTTACCAAACACAAAATGAGGAGCTTGAGCTTTTAAAAAGCACAAACACATCTTCAAAAA
GTTTTACCAAACCAAGCCTTAATCATTCACCCTCATAAATCAGGACACGTGTCAATGGAGAC
AAAAAAGAAAAAGCATTGATTAGAGTATTTGAGATTATCTTATCCTTGTCCATTTTTGTAAAA
TTCAAAGTATGATACGATTCTCACTATCACTATATATGAATAGAAAATGTGTAGAACCATCAA
AGTATGGCCCACAAGAATGGACCACAGCTTAAAAGAAAAGTGGTAATAGCAGAAGCCAAT
AGATAGAATCATAGAATATACCAAACAAAATTTGAGCAATTGAACATAGCAAATTGGCATAT
CCCTTATTATATTAATTCATGTCACTTTTCTGTGTGCCACCACATTCAACGTGCCATTAAAAA
GCTCATAGATTATGCATGACACATTCATCATCACCCTCCTTTTATTACTTCCTATATAAACCAA
ACTAGCCTTAACCTTAAGACTTCATTCAGAAGCAAAAAGCACAAAAAAGTAAAAGCTAAA
GGCTCTTATTGTTTTATCATCTAAGAAA

$P_{Ah\text{-}WT3AEF}$

CACCGTATCCAACAAATTCTCAAAAAGAACCTAAACAGATATTATTTACTATAATATAAGAAT
CAATTCTTTGAATCCAAAAAATGACAGAAAATATTCCATCTTATTCTTATTCTTAGTTGTTATG
TTTAAAATAAATTATTATTAATTATTAAAATAAAATATTATCAAAATACTTTATATAATTAAAAC
AACATTAAAACAGTGAACAAAGTTAGTTTGTGCATTGTTTAATAATTTCTTTATATTTTATATA
TGTGTTTTCTTTTGTCTAACAAAAATTTAAAATTAGTGAGTTTGTGTTAGTTACATTCGTGTT
AATGTTAGCGTTTGTATAACATAACTTGAGAGGAAAAGAAAACTTTTTAAAAGAAATCAAA
ACAATTATATTGATTGTTGAAAAATTACGAATAAATAAAGTGCCTTAATTAACTTAACCTGCA
TCAGTCCCTTGTACTATATATCTAGATGCATATTAATTATTATGATATATTGACTCGATCCCAAT
GATTCTCATGCTTTAATTTCGTTCCTTTGATTAGTACTATTTTTAGTTTCTATAATTTTTTTTCG
TTTAGTTCAATTCTTTTTGTTGTTGGGATGAGGAGTGCCCATGGTTTTCTAGCCTTCTCTCTG
GACTAAACCGCTGGCTTCTCCTACTGTTGCCATTGCGGATTAAAGAAAAATGTGTACTTTCT
TTAACTTGCTAAATTGATATCAAGAAAACAAAGGATGTGTGTGAGGTTCAATGGAAGAAAA
TAAAGGGATCTTTTTTATATGACTTTTGTGATGTTTGAGACACGAGAGGATAATGTCAGTATA
TATGTTTAATGTTAGTATTTGTTATATATATATATATATATATATATATATATAATTATTAAAAATGA
ATCCTCTTAATTTTTTTTTAATAATTGAAAGAGTAAAGTGTGATTATTTACTATTAATTTTATAA
ATAAAATCAAGAATAAATATGAGAGAAAAAAATAATGAAGAGTTAAAGATACACTTTATTAAT
TTATATATATAATTAGATATTAATTTAGTTAAATATATCAAATCATCTAACCATCCTTAAATATAA
ACTTCATATTATATGGATTTTTAACTTCTTTTTAATTTATTATTAGATCAAAGTTTTAGCATCTC
CAACAATTTTATAGTTAAATGAAAAAATTCTAATTAATTAAATAATATAGATTTTTAATTTTTTT
CTTAAAAAAAGAGAATAATAAAATTATGAATTATATAAAAAATACTGATATCAACATAAACTT
TGTTAGATTGCATTAGTTTTAGAACGAGTAATCTTACATATTCAGAAAATATTATTATTTTTTAT
TAGTATTTAGTTAATAATAATTTATACTAATATTTATAGGAGTTTTATACACAAAAAACATATAG
TTTATATCTATATTTATTAGAGTTTTATACACATAAATTAACACAATTTATACCTATACTTTTTTA
GAATTTATACACCTAAATTAATAAAATTTATCTGTTAAAAATAATTTAATATTTATAATGGTCAA
ATAATGACAAAAAATATTAAAACTTGTTAGTCTAAAAAAGATTCTCTTTTAAAATTTTATTTT

AGTAGAAAAGTTTTTGAAATTTTTATTTTAATAAATAAAAAATAAAAGTATTAAAAACTTCAA
TTTTACATATTTCCATAAAAGAAATTTCAATTAAAGCACGTGTTAACCAGACATTTGATTTTA
TTCGTTTAAACAAGATTAATTTGAATTACAACTTTATAAAGTAGATGATAATGCATTAATTTTT
GGGAAGACAAAAGAAATGAAGGGACAAGGACAAGGACTTAAATGAAAAACCACTGCCTG
AGACGGTGGATTTAGGGAGAAACCATCAAACTTCACCTGCCAAGGACGGGTGCAATGCATA
ATGCATGCACGATGCAACTCCCTCTCTAATTGTGTAATAAAAATAGATAAAGTAGTTAAATCA
TAAAATATGTGTTAATATTTTATTTTTTATATTGTCTACTAAGTTATTAATGTATATTTTTCTCTA
ATTAAAAAGAGTGTGATATTATGTAAAATGATTTATTGTAAGAAATAAAATATATATTATTTAG
AGAAAAAACTCAAATGTAGTTATTTTTATGTGAAGTTGTTAATTAAAAATTGTTAAATAATTT
AATATATTTGACTAAATTATAATATAACGTTTCTCAACTAACAATTTTAACTACATGTGAATTT
TTAACGTATTATTTATTAAAACAAGAAATATATTAGTAAATGATGGTCTAGGGAGCAGCTCAG
TTATAAATATGGCAGCAACTTAAACCCTCTCTTCACTCAAATTAAAGCTATTGAGTTATATATA
TTGTATTGCTCTGCTTCCTAAAATTGAACTTCTATTATTCTTCCATTAATGTTATACATTTTTTC
TTGAATAAAAGTAAGATATATAATATCTGAAT

$P_{Ah-I20Q6X}$

CAAGCGAATAATCCTAAAAGGCGCAAATTATAGATAAAAGCTGAAATGAGATTGGAAACAA
ATTCAAGAAATTGAGAGCAGAATAATTGAAGTACCGGGAGGAGAGGAGGTTATCGGCGAC
GAAGGCGGAGGAGGAGCAACGGCAGGGTTCGAAGAAGTGCTTTGGAGATTTGTGGAAGC
GTTGGTAGAAGAGGCGCGTGAAGAAGGTTTATTGAGGCGAGGTTTAGAAGAAGTAGAAGA
AGGAGGTTTGGATTTGGGTTTGGGTTTGATAAGGTTTGTGGAAATTGGAGAAGAAGAGTTA
AGAGTGTTGGTGCGATCGGATAGAGGGTTTCTGCGTTTAGTGCTTCGGTCAGCTTCCATCTT
TAGATCTTCGGGAACTCGGTTCCCATTTCAATTGAAGAAGAAGAAGAAGTAGTGATGC
GATGATTGAAAATGGAAGATTGAAGAAGCTGCTTGTTTGTTCGTTGAGTGTGTAGTGTTTTC
TCGTCTTTCCTGAATAGCGGGAATTTTCAGTTGAACAAGAAGGATTAAAAATCCTGAGGCA
AGCTTCCAGAGGAAGACTCCAATAGTCCACTGATTATAGTCAATGACAACGAACAGAAACC
TCAGCTGCTTTTTTTTTTTTTGGTAATATAGTGTCTTTTTGGACATGAATAAAGTTTTATATGGG
CCACTGATATAACACTCATACAAGCTGGGCCGATTCACATGTTTTAATACATGTGAATAGGCC
CAAAATAGGCCCATGGAAGAAAAACCAAATTGCTTTTTTTTTTCTAAGATATTATATTTCATT
GATAAAATGAGGACATTAGGACTGAGTTGAAAATTTTATTAAACTCTATCTTATCCTATTTGT
GGGTGGAGAATTTCTCAACCCTAACCCTACCCGCATTCTAAAATTCTAAACCCTAACATATC
CTATTCTATCCGCAAAAAATATCAAATTTTTTTAAAGTAAATATAAAATTCAATCATTTTGAAT
TTTATACATATTAATAACATAAAAAATAAAAAATTAATGTTCTAAATTACTAAATTAACTAACT
AGTTTTAATGGTTGTTTACTTATTGTAAGTTATTACATAAAAAACGTTATGGGTTCAACTCTC
ATTTCCTTCATTATAGGGTAGAGTAGGGTGCGGATTTTAGATGAGATTCTAGCTCAATATAGT
GCCTAATATTTATTTATAAAATTTAAAAATAAAGGGATGAGTCTCACTTTATTCTATCTCTATTT
TTAGATAGGTTAGACAAAAAAAAATTTATAAGTACCAAAAAATCTACTTTATTTTATATTATTCA
TTGTTTGACATGTCCGTAATACAAATTTATTAATCTTTTAGATCATGAGTAATTTTTTATAGCT
GCTTTAAAAATTTAACTTAAAATAAAAAGAATACAATTTTATTGCTCACATCTTAAATAATGA
ATGACAGAGAACAAACGGCTTGATTTTTCCTTGAAAATTTTGTCTTTTTTATTTTTTATTTTTT
TAATTTCTCTACTACCGGAAATGTGGAGATTAGTTGTATTTTGAGTATGAAAAATGTAGTAAA
ATTATCCCTTCTTGAGATTGATAACCGTTTTCAAATCTCAACCTGTACTTGGTGGTAGTTTAA
TGAATCTAATGCATGGACGGTGCCTACTCAGAAAAAAAAGGTATATGATTATAAATAATTAAAT
ATAACCCACAGGTCACAGATATAAATGGTGCTCATTTTTCCTTAACTTGATGCAGCACACAG
TCACAGCCTCAAAACTCAAAAGCCACTTTGTTTTTTATCAGTTTTTATGTTAAAAAGCTATTT

TCACATGGAAACATAAGAAAATCATAAGGCACGTGCATAAAAATCAGTTACTCAAAGTAATT
TAGTTGAGACTGAGAAGAGCAGAGACAAAACCGGGTTAAGCCTCTAGTTTAACCACTTTTC
TGTGTTCCAACAAACTAAAGGTGTGACCCAACACACTAAAGTCTAAACCCGCTAACACAA
ACAAACCCAGGGGCCACATTTATATAACATTTCAATTAATTAATAATTTAATTTCACCTACTAT
ATAAGTAGTATATCTACTACTCTTTCCTTTCACAATAACACTATACAACACAAATAAC

$P_{Ah\text{-}ELJ55T}$

TGTGGTTTGTTTGGGACTCTTTTATACCTCATTTGATTATAATGGAACTATTTTATTGGTCTTG
GCGATTCGTGGTTTTTACCTCTCACATTGAGAGAGGTTTTCTACGTTAAAATCTCGATGTATT
CTTGTTATAGCTTTACTTGCTATATTTGCTTGTTCATAGTACTGTCATATTGTGTTTAAGAGTAT
TTTCATATTATTCTTGTGTTGGATATTTGTGTTATTTTTATTGCTAAGCTCTTTCACAATTCAAT
ATATATATATATAAAATACGTATTTTCGGATATTGTACAAAGATTATTATTAATAAATATATTAAA
AGCTTAAATTTGAATAATCTTTATAAAAAAATTAATTTGCAATCTTAATATATATTTTAACACAT
ATTAGTTAAGTATTTATTTATTTATTGAGCATAGCAATCATTGTTAATTGTTAATGTTGGTTAAT
GGCATTTGTTAAGCCCCCTATGAACACATTGCTAGAATTAAAATGCAGCCCATGGCTTTGTA
AGAAAACAATTGGCATTTGTAATCGACAACCTTGAACAACCTTTATTACTTAGAAATAACCA
ACTCTTTTGTCAAAAACCATGTGCTCTCAAAAATTCCAATCACTCTTTTTCAAACAAGACCC
TTCATTCATGCAATTTCAATATGCGATCTTTGAATTGATTCAAGGGGATGTAGTGGACTACAA
ATCAAAGTTTAGATCATTGGATACGCCAGAATAACATTTAAAAATATAAATTTTTAAAAGAAA
TAATGTATAATACGATATAAAAAATTTTATAAATAAAAAAAAATTTATTTAACATTTTTGTCTT
TTAGAAGATATACTTCTTGTTCTTGATCTATAATATTTAAGAAGAATTTTAAAGGTACCAAATA
CACCGTTAATTCAATGATTTTAATGGTTGATTTTAATTATAAAAATATATATAATATATATTATTT
AAAATGAATGGTTAAGATTACTAGAATACTAGTGTTTTGGATGCATTTGAAAACTTTCTTAAT
ATTTATACATGATTATCATGGAATGGATGTGAATGTGTAATTATTTTAATTTTGATACACTGATA
GTGTAAAATATTTTATACCTTCATACAATTACGTTTTTTAATTTTATTATGAAGAAGGTAATGG
TGTAAGACAACAATATGGAGAAGCTAGTGCTTTACAGAGGCATATGGTGTCAAAGAGTTAC
TAATGGGACAGTGCGAATGCTCCCAAACAATCGAACAGTCCGATTTCAAAAAAAAATTAT
AATAATCAATTCGGACCATTCAATTTGATTTTCGTTTTATTAAAAAAAATTTCATACAACACA
GACAGTTCAATTTGCTTTTTTCAAAATTTAAAATTTTTCTCCTACAAATCACACCATCCGATT
TGTCTATAATATTTTTTTTTTAAACATAATTCGCATGTTCCGACTTGTCTATAGCACAAATAAA
AAAAAGATTACCCCCACAAATTAGTTTAATACCACTATATTCCATAAAACAGCACATATATGC
CTCCAATAACAAAAGATGAGCCCATGCAATTACAACAATTTTTTTAAATGATGAATCAATATA
AATGAATCAATATAAAAATAATTATTTTTACTAATACGAATGCATACGCTAATAAAACAATTTT
ACACCCTTTAAAATATAAAAACTTATATTTAAGATAAGGAGCACAAGAGTCTCATGAAAGCC
TCAACTAAATATCAAATCTCAGCCATCCATAATATCTTTCTCTTTTCCACCAGCAACGGTCGA
CATTCAATGTCCCTTCCAATTCCGCCACGTGTCAAGCCCCTCTATATATATACCTCGCCTCTCC
CTCCACTTTCTTTTTCACTGTCATTTTCTCGGGAAAACTGAGGGAGTGAGAAAACCGTGAA
AATTTTGAAAAACCACTGCGATTCACTGAACAACG

$P_{Ah\text{-}N9CMH4}$

TTAAAGCTCTTACTTCTTTAGGAGTTTAAAAGATGATTCTTAAAACAAGAGAATTAAATAAA
CAATCTTATACTTTCATAGCTATGTGACGAAACTTAAGAGGAAAAAGAGAGAAAAAAATTC
TCTCACCTTTTAAGTTTAAAATTTGTGATTTTCTACTCTTTGAGGATTAAGAACATGATTCCT
AAACAAGAAAAGATAAAAAAATTAAAATAGATCATCCATGGCAATTTTCTCATGGCCGAATT
TATGTGAAGGAGAAAGAAGAGAATTTTTTTGACTAACCTTGATTAAATTGGTATGGATGAGA

AGAAAATTAAGACAGAAATTAATTAATTGGAATAGAATTTGGATTACAGCCTAAAATCCTTC
GTAAATCAAGCATGAATTTTAGATGAGCATATTTGGTTCTCTCTTTTCTCTCATATTTCTTATG
AAAATAAGGAAGAAAATAAATTATAATAAATGAGAGAAACAGAAAAAGTCTATGAAAATAA
AGATAAAAGGATAAGATTGTCGCTCAAAATATTTCTTAATTAAGAGATAATTATCAAGTGTGG
TAATTATTTTACATGATTTTATTTTATCTGGTCAATTAGTTCTTTTATTAGTGTCAATTATATTGA
TATGATAAATTATCTCAACTCTAAAATAACTCTAAAATATTTTGGTATAGTTTACTAGAACAAA
GAATATACTAATAAAATTCTCTTACTAGTTAAATATTAATCCGATAATTATGTAGTCTAAAAAA
AATCTTTAATTATTCACAAATTAATATTATTTTTACAAATAATAATTTTAAATATTTTTATTTTTG
ATACGGGCTTCACTCATTAAATCGAATTAATTTGCGATACACGATTTTTTTAGATTCTGATCAt
AGAAAATTCTAAAATACGAAATATTGGATTGATACTTCAACAATATGAAAAATTTATGAATTT
GAAGTACTTTTCTTAAAGTACCCATTAATGTTAATTATAAATTATGAGAAAAAAATGGACATA
AAATTATATTTGATTCTGAGAATAAAATAAGATAAAACACTAAAAATAAAACATAAAGGATA
GAGATACAAAAATTAGTGTTCTAGGATTTTGTTTAGTAATAAACTAGAACAAATTATGAAAG
TCTAATTTATTCTATTTTTTATTAAAAAATTTGAAAAAAATATAATTATAAAAAATTAATAAAA
ATAATAAAAAAAATAAAAAATAAGTTGTCTCTGTGTGTTCTAATTGATAAGATAAACACAAA
ATATACTAATTTAATATTTTTAGACACAACATCTCTATTTATATCTCATCTATCAAATATTGTGTT
CCTATGTCTATATCTTAGTGTCTTGTACCTATATACACGAACATAGCGCAACCTAATATACTTG
TTGGTAGTCACTAAAAAATTAATTTAGATTCTCTAAATTTTAGATTTTTATTTTAGAGGATAAA
GTAAAATCTCTCACCAATTTTTTCTTTCATGTATTTTTTTGTCCCACCTATTAAATAAATAAGA
TAGATCATACTTTATCTTCTAAAATTAAATTTAAAATTTAAAAAATTCAAATCCCTAAAAAATA
AATTAAATTAAAGTTTGTAGACCGATTTTTTTATTCAGATCTTTCATTTTTAGAAACTTATAAA
GTTCTTCTCTTAAGCTCCAATCAATGAACCTACAAAACCCTTTAAATTGTATCTCTGATTCAA
TCCCAGTATTGTAGACATTCCCGCATTTTCACCCCAATCATTTTTTATTTTCCCCTTGTATTTTT
TAGAAAACCATTTATCTCCGCCTATTTTCATTATAAAGGCATCCGTTGACAGCATTTATGTACT
TTGTACCCTTCCATGATATGAAATTATGGATACATTATTTGAATTCTTTCTTGCATAAGATAAT
GTTGTTTCTGCATGCAATCAGCGAAACCCATGTTCATACTTTCAACTTTGCTACCATTGATTT
GAAAACAAGTTTCCAGAAGCCACCTTTTGCAATAACCAAGCAACATGTATAGACATATTACT
TCTATAAATAACAGGCGAAACATTGAATTGCTACATCAATTAGCTCTAATAATAATAACTCGA
CTTGATTTCTATACGAGAATTTAAGT
